# From Structure to Dynamics: Activation Mechanism of the G Protein-Coupled Bile Acid Receptor 1‑G_s_ Complex

**DOI:** 10.1021/acsomega.6c02725

**Published:** 2026-08-13

**Authors:** Bianca Fiorillo, Federica Moraca, Francesco Saverio Di Leva, Valentina Sepe, Stefano Fiorucci, Vittorio Limongelli, Angela Zampella, Bruno Catalanotti

**Affiliations:** † Department of Pharmacy, 9307 University of Naples “Federico II”, via D. Montesano, 49, 80131 Naples, Italy; ‡ Department of Medicine and Surgery, University of Perugia, Piazza L. Severi, 06132 Perugia, Italy; § Faculty of Biomedical Sciences, Euler Institute, 27216 Università della Svizzera italiana (USI), via G. Buffi, 13, CH-6900 Lugano, Switzerland

## Abstract

The G protein-coupled bile acid receptor 1 (GPBAR1, also known as TGR5) is a key mediator of bile acid signaling, exerting its physiological effects through coupling with the stimulatory G protein (G_s_). This interaction stabilizes the receptor’s active state and triggers downstream signaling; however, the dynamic features underlying GPBAR1 ligand recognition, activation, and G protein engagement remain poorly understood. In this study, we investigated the molecular basis of GPBAR1 receptor activation by combining microsecond-scale molecular dynamics simulations, principal component analysis, and network-based allosteric communication analysis, using lithocholic acid (LCA), the most potent endogenous agonist, as a reference compound. By comparing LCA-bound and ligand-free GPBAR1 in the presence and in the absence of G_s_, we disentangled the respective contributions of agonist binding and G protein coupling to receptor activation. Our results show that LCA promotes activation-associated rearrangements of TM5, TM6, and key microswitches, reshaping the GPBAR1 conformational landscape independently of G protein recruitment. These ligand-induced changes strengthen the coupling interface with the α5 helix of Gα_s_ and facilitate allosteric communication between the orthosteric and intracellular sites. While agonist binding is sufficient to trigger these rearrangements, G_s_ coupling further stabilizes more organized active-like conformational ensembles. Overall, our findings provide a dynamic mechanistic view of LCA-driven GPBAR1 activation and G-protein engagement, highlighting its role as a molecular effector of bile acid signaling and offering molecular details relevant to GPBAR1-targeted drug discovery.

## Graphical abstract

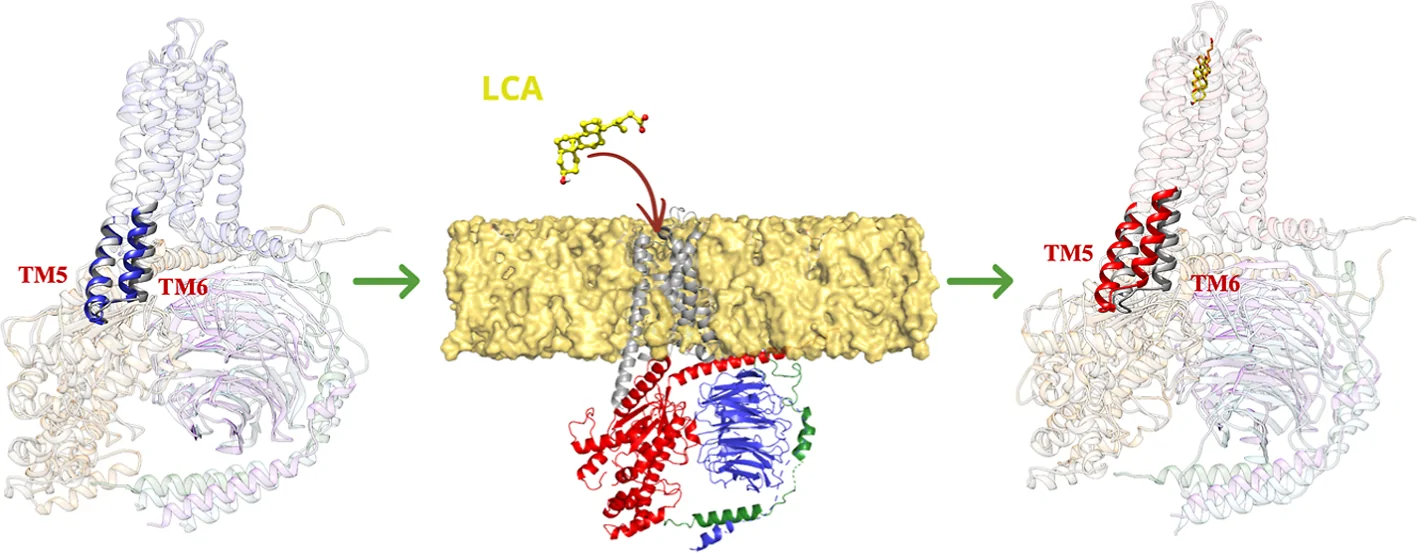

## 1 Introduction

The G protein-coupled bile acid receptor 1 (GPBAR1), also known as TGR5, is a class A G protein-coupled receptor (GPCR) that acts as a membrane-bound sensor for bile acids, integrating metabolic and immune signaling across multiple physiological systems. GPBAR1 is expressed in a variety of tissues, including enteroendocrine cells, hepatocytes, brown adipose tissue, skeletal muscle, and immune cells such as macrophages and monocytes. Upon activation by endogenous bile acids, GPBAR1 preferentially couples to the stimulatory G protein G_s_, leading to increased intracellular levels of cyclic AMP (cAMP) and subsequent activation of protein kinase A (PKA)-dependent pathways that regulate gene transcription and cellular function.^1,2^ This receptor has emerged as a promising pharmacological target in recent years, due to its central role in modulating glucose and lipid metabolism, energy expenditure, intestinal hormone secretion, and inflammatory responses.^2–6^

In the gastrointestinal tract, the activation of GPBAR1 on enteroendocrine L cells promotes the release of incretin hormones, particularly glucagon-like peptide-1 (GLP-1), thereby enhancing insulin secretion and contributing to glycemic control.^2,5,7,8^ In brown adipose tissue and skeletal muscle, GPBAR1 signaling promotes mitochondrial activity and thermogenesis, supporting increased energy expenditure and lipid mobilization, which are beneficial in the context of obesity and type 2 diabetes.^9^ Finally, in immune cells, particularly monocytes and macrophages, GPBAR1 activation has been shown to suppress pro-inflammatory cytokine production and shift macrophage polarization toward an anti-inflammatory (M2-like) phenotype, revealing potential for therapeutic modulation in chronic inflammatory conditions such as inflammatory bowel disease (IBD).^10,11^

Altered expression or signaling through GPBAR1 has been implicated in the pathogenesis of several diseases. Reduced receptor activity, for example, has been associated with insulin resistance and impaired glucose tolerance, while aberrant or constitutive activation may be involved in the progression of certain cancers, such as cholangiocarcinoma, where bile acid signaling intersects with oncogenic pathways.^6,12,13^ These observations highlight the importance of gaining a detailed molecular understanding of GPBAR1 activation, ligand binding, and downstream effector coupling in both the physiological and pathological contexts.

Endogenous GPBAR1 ligands represent a diverse family of bile acids, and it is their structural variability that underpins differential agonistic efficacy and tissue-selective pharmacology. Beyond their physiological roles, these compounds are increasingly recognized as privileged scaffolds for drug design, particularly in the treatment of metabolic and inflammatory disorders. Among endogenous bile acids, lithocholic acid (LCA) (Figure 1) is the most potent endogenous GPBAR1 agonist (EC_50_ = 0.53 μM),^14^ displaying a high binding affinity and robust G_s_-mediated signaling.

**Figure 1:**
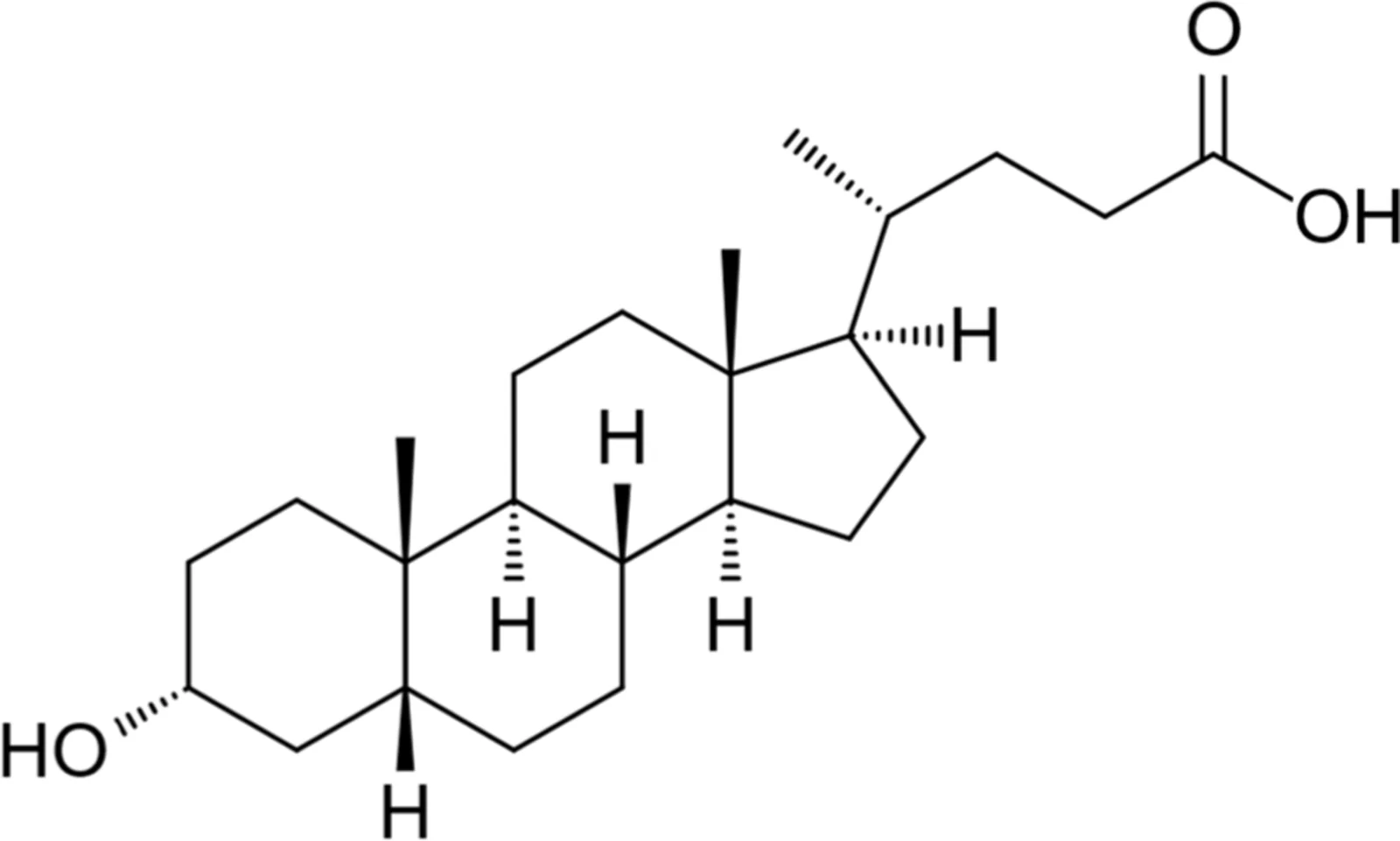
Two-dimensional (2D) chemical structure of LCA.

LCA represents a widely used reference agonist ligand for GPBAR1 because of its high potency, well-characterized pharmacological profile, and extensive use in experimental and computational studies of this receptor.^14–16^ In this study, LCA was used to investigate the molecular factors that trigger receptor activation, given that it represents the most potent endogenous agonist of GPBAR1 among naturally occurring bile acids, with an EC_50_ of 0.53 μM, compared to 1.0, 4.4, and 7.7 μM for DCA, CDCA, and CA, respectively.^14^ This higher potency facilitated the characterization of ligand-bound receptor conformations during molecular dynamics (MD) simulations. Until recently, the lack of experimentally determined GPBAR1 structures severely limited a mechanistic understanding of bile acid recognition and receptor activation. In this context, homology modeling and other computational approaches, together with structure–activity relationship (SAR) and site-directed mutagenesis studies, provided the primary framework for interpreting ligand binding and receptor activation.^17–19^ Early models, such as that developed by D’Amore et al.,^20^ exploited the conservation of class A GPCRs to predict active-like receptor conformations and rationalize bile acid binding modes, guiding docking, virtual screening, and mutagenesis experiments. In particular, these studies suggested that subtle differences in hydroxylation patterns and side-chain flexibility translate into distinct receptor engagement modes and downstream signaling biases^21,22^ and identified key residues within TM3, TM5, TM6, and TM7 as important for ligand recognition. However, in the absence of direct structural information, how different agonists stabilize specific active-state conformations and how these arrangements couple to G_s_ remained largely unresolved.

A breakthrough came with the publication of cryo-electron microscopy (cryo-EM) structures of GPBAR1 in complex with the G_s_ protein, including the INT-777-bound assembly (PDB ID: 7CFN),^21^ which offered the first high-resolution structure of the receptor active state. Nevertheless, such a structure lacks flexible loops, the terminal regions of GPBAR1 and Gα_s_, and the entire α-helical domain (AHD) of Gα_s_, which is critical for nucleotide exchange. Furthermore, cryo-EM models reproduce a static representation of the protein that does not capture the dynamic interplay between the receptor, the G protein, and the surrounding lipid bilayer. This aspect is of paramount relevance in modern medicinal chemistry, encompassing efforts to connect ligand binding with GPCR functional dynamics, particularly through dynamic structure–activity relationship (dySAR) studies,^23^ as well as the development of molecular glues and allosteric modulators that target the receptor-G protein interface to modulate transducer signaling.^24,25^ To address these limitations, computational modeling is required to extend experimental structures into functionally complete and dynamically tractable systems.

In this study, we constructed and refined a full-length GPBAR1-Gα_s_β_1_γ_2_ model based on the INT-777-bound cryo-EM structure assembly (PDB ID: 7CFN),^21^ embedded in a lipid bilayer and encompassing the native post-translational modification of N-linked glycosylation at Asn76^ECL1^ (superscripts refer to Ballesteros-Weinstein numbering),^26,27^ to mimic a physiologically relevant membrane-associated state. Using this model, we investigated how the endogenous agonist LCA interacts with the orthosteric site and modulates the conformational landscape of GPBAR1 and its coupling to G_s_. By comparing LCA-bound and ligand-free microsecond-scale MD simulations both in the presence and in the absence of G_s_, we characterized how agonist binding and G protein coupling modulate receptor flexibility, microswitch organization, TM5/TM6 rearrangements, and long-range allosteric communication between the ligand-binding pocket and the intracellular G protein interface.

Despite the increasing availability of GPBAR1 structural information, the dynamic mechanisms linking bile acid recognition to receptor activation and G-protein engagement remain incompletely understood. Addressing these aspects is particularly relevant, given the structural and pharmacological peculiarities of GPBAR1 relative to more extensively characterized class A GPCRs. Our computational analysis provides a dynamic perspective on how the endogenous ligand LCA reshapes GPBAR1 conformational ensembles and signaling-related structural features, prompting the rational design of synthetic agonists, allosteric modulators, or biased ligands with improved efficacy and selectivity for the treatment of metabolic and inflammatory diseases.

## 2 Methods

### 2.1 Comparative Modeling, Structure Refinement, and System Preparation

The cryo-EM structure of the INT-777-bound GPBAR1-Gα_s_β_1_γ_2_ complex (PDB ID: 7CFN)^21^ was selected as the starting template because it represents an active-state GPBAR1 conformation bound to a steroidal agonist structurally related to LCA. Alternative GPBAR1 cryo-EM structures were also evaluated (Table S1). However, 7CFN was considered the most suitable template for the present study owing to the similarity between INT-777 and LCA and the compatibility of the orthosteric binding site with bile acid recognition.

Prior to model building, nanobody fragments, the ligand INT-777, cholesterol, palmitic acid, and water molecules were removed. Missing segments in both the receptor and G protein subunits, which were unresolved in the experimental structure, were modeled using MODELLER version 10.6,^28^ based on predicted coordinates retrieved from the AlphaFold database (https://alphafold.ebi.ac.uk).^29^ In particular, the GPBAR1 sequence was aligned to the AlphaFold-predicted active-state structure, and the missing regions Ile13^N‑ter^-Ala17^1.35^ and Thr288^8.51^-Ala306^C‑ter^ were reconstructed. For the Gα subunit, the Met1-Lys8, Arg61-Thr204, and Asn254-Thr263 segments were derived from the AlphaFold-predicted model, after alignment on the Ras domain backbone. For Gβ, the N-terminal residue Met1 was modeled ab initio. Similarly, for Gγ, the N-terminal (Met1-Asn4) and C-terminal (Glu63-Leu71) fragments were reconstructed ab initio. Predicted secondary structure elements were incorporated to refine poorly conserved regions, and the resulting models were ranked according to their discrete optimized protein energy (DOPE) score. The model with the lowest DOPE score was selected and structurally validated via Ramachandran plot and MolProbity analysis,^30^ showing >95% of residues in favored regions and Z-scores (−1.43 ± 0.24) comparable to experimentally solved GPCR-G_s_ complexes. Importantly, reconstruction of the previously unresolved regions restored the complete architecture of the signaling complex, including the AHD of Gα_s_, which is absent from the experimental cryo-EM structure and plays a central role in nucleotide exchange. The final full-length GPBAR1-Gα_s_β_1_γ_2_ model thus provided a structurally coherent framework suitable for docking and MD studies. The resulting model was then processed using the Protein Preparation Wizard implemented in Maestro software (Schrödinger Suite 2022–4),^31^ which involved the assignment of proper bond orders, the addition of hydrogens, the optimization of protonation states at physiological pH, and the minimization of the structure to relieve steric clashes. Gα_s_β_1_γ_2_ heterotrimer was removed from the LCA-bound GPBAR1-Gα_s_β_1_γ_2_ complex (aka LCA:GPBAR1:G_s_) and the ligand-free GPBAR1-Gα_s_β_1_γ_2_ complex (aka GPBAR1:G_s_) to generate the starting configurations for the LCA-bound GPBAR1 receptor in the absence of G protein (aka LCA:GPBAR1), and the ligand-free GPBAR1 receptor in the absence of G protein (i.e., receptor-only; aka roGPBAR1) calculations.

### 2.2 Ligand Preparation

The three-dimensional structure of LCA was generated using the graphical user interface of Maestro version 13.4.^31^ The protonation state at pH 7.4 in water was calculated using the Epik module.^32^ Finally, the ligand was minimized using the OPLS2005 force field^33^ using 2500 iterations steps of the Polak-Ribière Conjugate Gradient^34^ algorithm.

### 2.3 Docking Calculations

Docking of LCA to the GPBAR1 receptor was performed using the standard precision docking protocol implemented in the Glide software package^35^ and the OPLS2005 force field.^33^ A grid box of 3 × 3 × 3 nm was defined around the ligand binding cavity of the GPBAR1 receptor. Ligand conformational sampling was enhanced by a factor of 4, and a total of 100 ligand conformations were generated within the receptor’s orthosteric site. The lowest energy pose in terms of both Glide Emodel and GlideScore was selected for further analysis.^36^ This pose exhibited a docking score of −6.691 kcal/mol and an orientation highly similar to that observed for INT-777 in the experimental structure (RMSD = 0.08 nm, computed on the steroidal scaffold) (PDB ID: 7CFN).^21^

### 2.4 MD Calculations

MD simulations were performed with the GROMACS suite version 2021.5,^37,44^ using the CHARMM36m force field for the proteins and lipids, and the CHARMM General Force Field (CGenFF) parameters for the ligand.^38,39^ The preparation of the four simulated systems (LCA:GPBAR1:G_s_, GPBAR1:G_s_, LCA:GPBAR1, and roGPBAR1) is described in Section 2.1. Each system was simulated to distinguish ligand-driven conformational effects from those stabilized by G protein engagement. All systems were embedded in a heterogeneous bilayer of size 16 × 16 nm, composed of 1-palmitoyl-2-oleoyl-*sn*-glycero-3-phosphocholine (POPC) and cholesterol (CHL) at a molar ratio of 7:3, using the Membrane Builder tool of the CHARMM-GUI web server (https://charmm-gui.org).^40,41^ Prior to membrane generation, the proteins were reoriented by alignment with the corresponding OPM-deposited structure (https://opm.phar.umich.edu).^42^ An N-linked glycosylation consisting of one N-acetylglucosamine (GlcNAc) moiety was added at Asn76^ECL1^.^27^ Additionally, a disulfide bond was introduced between Cys85^3.25^ and Cys155^ECL2^, in accordance with known GPBAR1 structural features. Both the N- and C-termini of the protein chains were capped at the points of truncation to mimic the native terminal environments. The resulting system was solvated with explicit TIP3P water and 0.150 M NaCl in a 13 × 13 × 17 nm box.

Energy minimization was performed using the steepest descent algorithm in a single-step procedure with restraints gradually released.

The system was equilibrated using a multistep protocol consisting of 16 sequential stages, designed to ensure a gradual relaxation of the structure prior to production simulations. Thermalization was carried out under an *NVT* ensemble by progressively heating the system from 0 to 300 K using the Berendsen thermostat with a coupling constant of 0.1 ps.^43^ Equilibration steps were performed under *NPT* conditions employing the Berendsen barostat with semi-isotropic pressure coupling at 1 bar, allowing the system density and simulation box dimensions to stabilize.

The leapfrog integration algorithm was used throughout, with all bonds involving hydrogen atoms constrained using the LINCS algorithm.^44,45^ A time step of 1 fs was employed during the initial heating phases and increased to 2 fs during the later equilibration stages. The total equilibration time amounted to approximately 18 ns, after which the system was considered to be stable in terms of temperature, pressure, and density.

Production simulations were then performed in the *NPT* ensemble using a time step of 2 fs. Temperature was maintained at 300 K using the Nosé-Hoover thermostat,^46^ while pressure was controlled at 1 bar with the Parrinello–Rahman barostat under semi-isotropic coupling.^47^ Periodic boundary conditions were applied in all directions, and long-range electrostatic interactions were treated using the particle-mesh Ewald method. Each production run was carried out for 500 ns. For each of the four systems, three independent replicas were performed, resulting in an overall sampling time of 1.5 μs per system. Analysis of MD simulations was performed after merging the three independent replicas and removing solvent and ions, with the exception of the radius of gyration, which was calculated on the 500 ns replicas of each system independently. Trajectory clustering was performed using the GROMACS gmx cluster tool with the GROMOS method,^48^ employing a 0.15 nm cutoff and with a stride of 100. RMSD, root mean square fluctuation (RMSF), and the radius of gyration were calculated with GROMACS gmx rmsd, rmsf, and gyrate tools, respectively,^37,44^ whereas distances and microswitch dynamics were monitored with the MDAnalysis tool.^49,50^

### 2.5 SIFt Analyses

Structural interaction fingerprint (SIFt) analyses^51^ were carried out on the merged MD trajectories using a Python implementation. Ligand–receptor interactions involving both backbone and side-chain atoms were encoded as a 7-bit fingerprint, followed by seven listed interaction types: hydrogen bonds with the protein acting as donor (Hbond_ProD) or acceptor (Hbond_ProA); apolar contacts defined by carbon–carbon proximity; aromatic interactions in face-to-face (Aro_F2F) and edge-to-face (Aro_E2F) geometries; electrostatic interactions with positively (Elec_ProP) or negatively (Elec_ProN) charged residues. Apolar interactions were identified using a distance cutoff of 4.5 Å, aromatic and electrostatic interactions using a cutoff of 4.0 Å, and hydrogen bonds using a cutoff of 3.5 Å. The probability of each interaction was estimated within a two-state Markov framework by sampling the posterior distribution of the transition matrix with standard Dirichlet priors, as previously described by Noé et al.^52^ The resulting interaction fingerprints were visualized and plotted using RStudio vers. 2025.05.1.

### 2.6 Principal Component Analysis

Principal component analysis (PCA)^53^ of the GPBAR1 systems was performed on TM5 and TM6 to investigate their collective motions in the presence and in the absence of G protein. This analysis was carried out using gmx covar and gmx anaeig. To enable direct comparison among systems, PCA was performed on the three trajectories concatenated, yielding 1.5 μs of sampling per system, and aligned to the receptor backbone. The trajectories were stripped of solvent and ions, and global translational and rotational motions were then removed by fitting the trajectories to the receptor backbone of the first MD frame.^37,44^ The analysis was focused on the residues forming TM5 and TM6. The coordinate covariance matrix was generated and diagonalized to obtain the PCs as eigenvectors and eigenvalues. The first four PCs were analyzed in detail. The dominant mode (eigenvector 1) describing the motions of TM5 and TM6 was subsequently visualized in PyMOL vers. 2.3.0, where the displacement vectors illustrate the magnitude and direction of the collective motions (see Movies S1 and S2).

### 2.7 Network-Based Analysis

Allosteric communication within GPBAR1 was analyzed using the MDpath software package,^54^ which identifies the most probable routes of dynamic information transfer between residues by combining atomic correlation analyses with graph theoretical approaches.

For each simulation, the receptor was extracted from the trajectories, and a residue–residue network was constructed based on the normalized mutual information (NMI) of atomic fluctuations. To ensure a consistent comparison among the four systems, the residues defined as “ligand-interacting” were selected from the centroid structure of the most populated receptor cluster obtained from the LCA:GPBAR1:G_s_ MD trajectory. These residues were in direct contact with LCA during the simulation’s most representative conformational state, and they were then used as the starting points of the allosteric network in the LCA:GPBAR1:G_s_, GPBAR1:G_s_, LCA:GPBAR1, and roGPBAR1 trajectory analyses.

The MDpath algorithm evaluates correlations between residues and constructs possible allosteric routes according to the user-defined geometric and statistical parameters. In our analysis, a distance cutoff of 0.6 nm was applied to define residue connectivity within the graph (graphdist flag), while 0.3 and 1.2 nm were used as the minimum and maximum thresholds for contact formation (closedist and fardist, respectively).

To smooth the resulting paths, a spline factor of 0.2 was adopted (spline flag), and 200 bootstrap resamples were performed (bs flag) to estimate the statistical confidence of each pathway. The top 30 most probable paths (numpath) were retained for further analysis and visualization.

To accelerate the computation of correlation matrices and clustering steps, each trajectory was processed using 16 CPU cores (CPU flag). The resulting NMI matrices were clustered, and the pathways with the highest confidence were extracted and ranked according to their occurrence probability.

The identified communication routes were finally mapped onto the receptor structure and visualized using PyMOL vers. 2.3.0 by connecting the Cα atoms of sequential residues along each path. This analysis identified the major allosteric communication networks connecting the ligand binding pocket to the intracellular domain and revealed how ligand binding reshapes the internal signaling architecture of GPBAR1. Path weight values for each residue were calculated from MDPath. For visualization, only residues whose cumulative path weight exceeded 0.5 were included, and the values were plotted as side-by-side bars for the ligand-bound and ligand-free systems using RStudio vers. 2025.05.1 and the ggplot2 package, with residue labels ordered according to the protein sequence.

MD trajectories were visualized using VMD software,^55^ and all figures were rendered using UCSF Chimera^56^ and PyMOL version 2.3.0 (https://pymol.org).

## 3 Results

### 3.1 The Dynamic Binding Mode of LCA Identifies Stabilizing Interactions

To evaluate the binding mode of LCA at the GPBAR1 orthosteric site, we initially performed docking calculations with Glide.^31^

The top-ranked docking pose (−6.69 kcal/mol) places the steroidal core of LCA deep within the transmembrane (TM) bundle (Figure 2), establishing hydrophobic interactions with residues from TM2, TM3, TM6, and TM7, such as Leu71^2.60^, Leu74^2.63^, Trp75^2.64^, Pro92^3.32^, Phe96^3.36^, Phe161^ECL2^, Leu244^6.55^, Val248^6.59^, Ala250^6.61^, Pro259^7.32^, Leu262^7.35^, Leu263^7.36^, and Leu266^7.39^. The terminal hydroxyl group is oriented toward Tyr240^6.51^ in TM6 and forms a hydrogen bond with its phenolic side chain. The presence of the 3-OH group in the α-configuration is known to be an important determinant of bile acid agonistic activity.^57^ The carboxylate tail, meanwhile, orients toward the extracellular vestibule, consistent with previously reported SAR data. Additional polar interactions are established with Tyr89^3.29^, Asn93^3.33^, Ser247^6.58^, and Tyr251^6.62^. Notably, this binding orientation closely mirrors that of INT-777 in the reference cryo-EM structure (RMSD = 0.08 nm, computed on the steroidal scaffold), further supporting the reliability of the docking results.

**Figure 2:**
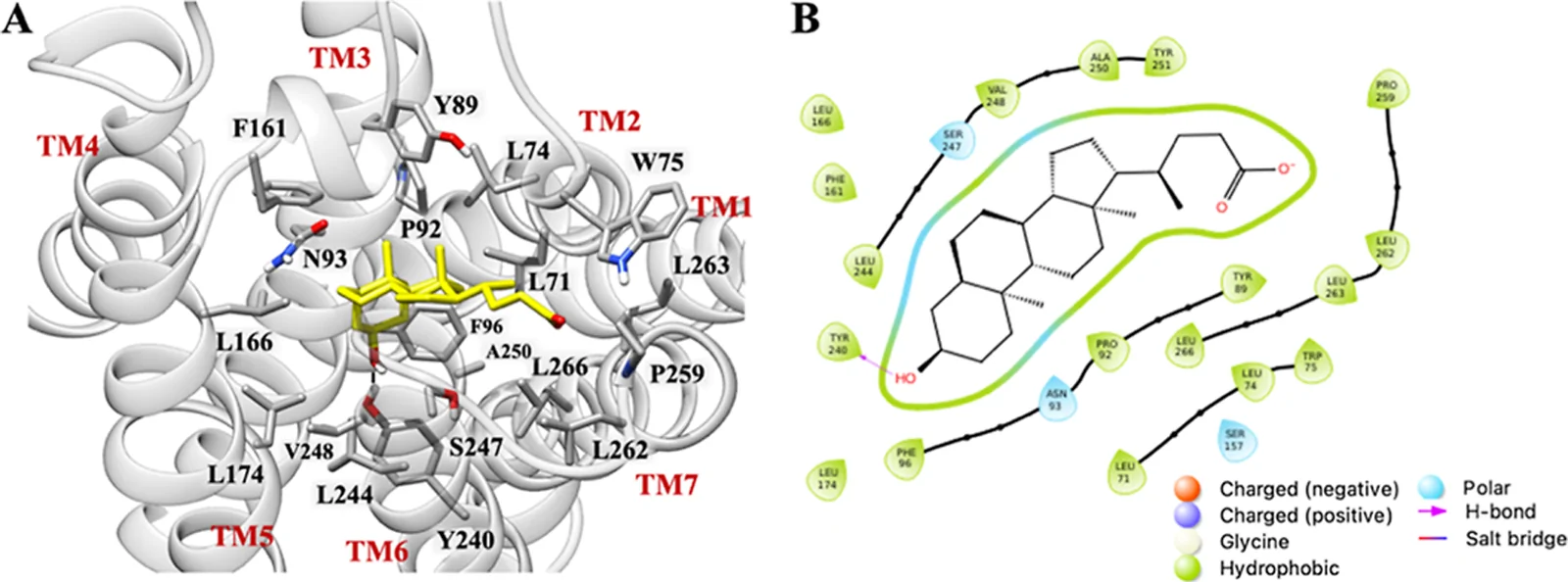
(A) Docking pose of LCA in the GPBAR1-Gα_s_β_1_γ_2_ model. The ligand is represented as yellow sticks, while the interacting residues of the receptor are shown as gray sticks and labeled; oxygen and nitrogen atoms are colored red and blue, respectively. The receptor is represented as ribbons with its helices labeled. H-bonds are displayed as black dashed lines. (B) Ligand interaction diagram (LID) of LCA in the GPBAR1-Gα_s_β_1_γ_2_ structural model.

To evaluate the stability and energetics of the predicted LCA pose and its impact on the conformational dynamics of GPBAR1, the LCA:GPBAR1:G_s_ complex was submitted to three independent 500 ns-long MD simulation replicas, in parallel with the GPBAR1:G_s_ assembly, thereby enhancing conformational sampling and improving statistical robustness. The inclusion of the G protein in both systems ensured that the receptors in GPBAR1:G_s_ and LCA:GPBAR1:G_s_ could be compared from structurally and functionally equivalent starting points. Each system was embedded in an explicit POPC/CHL lipid bilayer (molar ratio 7:3) and simulated under physiological ionic conditions to reproduce a native membrane environment.

Throughout the LCA:GPBAR1:G_s_ MD simulations, the LCA remained stably accommodated within the orthosteric pocket (Figure 3), with a slight deviation from the initial docking pose (average RMSD equal to 0.19 nm computed on the ligand’s heavy atoms) (Figure S1E′). This limited deviation indicates that the docking pose represents an energetically favorable binding mode that is preserved under dynamic conditions, supporting the robustness of the initial docking predictions.

**Figure 3:**
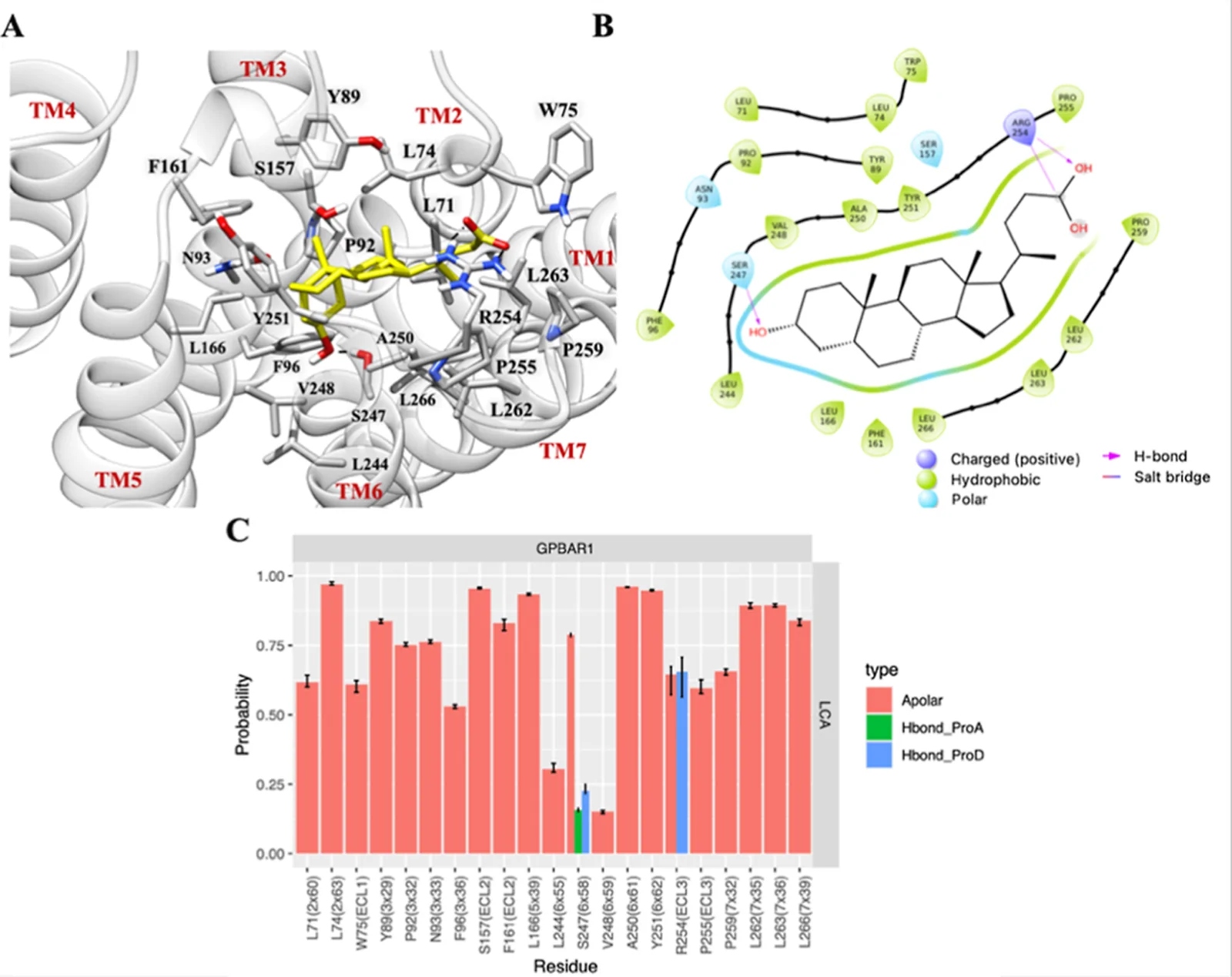
(A) Centroid conformation of the most populated cluster obtained from the three MD replicas of the LCA:GPBAR1:Gs complex. The ligand is represented as yellow sticks, while interacting residues of the receptor are shown in gray and labeled; oxygen and nitrogen atoms are colored red and blue, respectively. The receptor is shown as ribbons with helices labeled. H-bonds are displayed as black dashed lines. (B) LID of the MD-derived centroid of the most populated LCA binding mode in the GPBAR1-Gα_s_β_1_γ_2_ model. (C) SIFt analyses showing probabilities of ligand–receptor interactions formed during three 500 ns MD simulations of the LCA:GPBAR1:G_s_ system. Interaction types include apolar carbon–carbon contacts (Apolar, pink), hydrogen bonds with the protein as an acceptor (Hbond_ProA, green), and hydrogen bonds with the protein as a donor (Hbond_ProD, blue). Only interactions with an average probability greater than 10% are displayed.

The hydrophobic network involving the steroidal scaffold and residues in TM3, TM5, TM6, and the extracellular loops (ECLs) is largely preserved, maintaining the integrity of the ligand binding cavity. In particularly, the ligand’s core interacts with Leu71^2.60^, Leu74^2.63^, Trp75^2.64^, Tyr89^3.29^, Pro92^3.32^, Asn93^3.33^, Phe96^3.36^, Ser157^ECL2^, Phe161^ECL2^, Leu166^5.39^, Leu244^6.55^, Val248^6.59^, Ala250^6.61^, Tyr251^6.62^, Pro255^ECL3^, Pro259^7.32^, Leu262^7.35^, Leu263^7.36^, and Leu266^7.39^.

Consistent with docking results, the MD-derived binding poses align well with site-directed mutagenesis and SAR data, which identify several of these residues, including Asn93^3.33^, Leu244^6.55^, Leu263^7.36^, and Leu266^7.39^, as key determinants of bile acid recognition and GPBAR1 activation.^58–60^ Mutations at these positions, such as Leu^2.60^Ala, Trp^ECL1^Ala, Phe^3.36^Ala, Ser^45.52^Ala, Phe^ECL2^Ala, Leu^5.39^Ala, Leu^6.55^Ala, Tyr^6.62^Ala, and Leu^7.39^Ala, have been shown to markedly reduce ligand affinity and agonist efficacy, supporting their direct involvement in orthosteric recognition and signaling transduction.^21^

Together, these residues mostly participate in an extended hydrophobic interaction network that stabilizes LCA in a binding mode consistent with known functional determinants of GPBAR1, thereby reinforcing the mechanistic interpretation of the docking and MD results within the context of existing experimental evidence.^20,41,57,61–64^

In addition to TM helices, MD simulations reveal a significant contribution of ECL residues to ligand stabilization. In particular, as confirmed by the SIFt analysis (Figure 3C), polar interactions between the LCA carboxylate group and Arg254^ECL3^, together with hydrogen bonding to Ser247^6.58^, suggested that extracellular regions contribute not only to ligand recognition but also to the dynamic stabilization of the bound agonist. Taken together, these data indicate that ECLs shape the ligand-binding vestibule and regulate access to the orthosteric site as observed in other class A GPCRs,^65^ acting as a dynamic gate that stabilizes ligand residence and enhances receptor engagement and signaling efficacy.

To investigate the contribution of G protein coupling to ligand stabilization, the binding mode of LCA was also analyzed in the LCA:GPBAR1 (G-protein-free) system. Throughout the simulations, LCA remained stably accommodated within the orthosteric pocket (average RMSD equal to 0.17 nm computed on the ligand’s heavy atoms) (Figure S1F′), preserving the overall orientation observed in the LCA:GPBAR1:G_s_ complex (Figure 4A,B). The steroidal scaffold remained deeply buried within the TM cavity and maintained the extensive hydrophobic interaction network observed in the LCA:GPBAR1:G_s_ system, including contacts with residues from TM3, TM5, TM6, TM7, and ECL2. Only minor differences were detected in the organization of the interaction network, with additional contributions from Tyr240^6.51^ and Thr243^6.54^ and a hydrogen bond between the ligand carboxyl group and Gln253^ECL3^. Furthermore, Ser247^6.58^ formed a persistent polar interaction with the ligand hydroxyl group. Interestingly, the interaction probability associated with Ser247^6.58^ was higher in the receptor-only simulations than in the corresponding LCA:GPBAR1:G_s_ complex. Rather than reflecting enhanced receptor activation, this behavior likely results from a redistribution of local polar interactions within the orthosteric pocket. In the absence of G protein coupling, Ser247^6.58^ becomes a more persistent interaction partner of the ligand hydroxyl group, partially compensating for the reduced contribution of other extracellular polar contacts.

**Figure 4:**
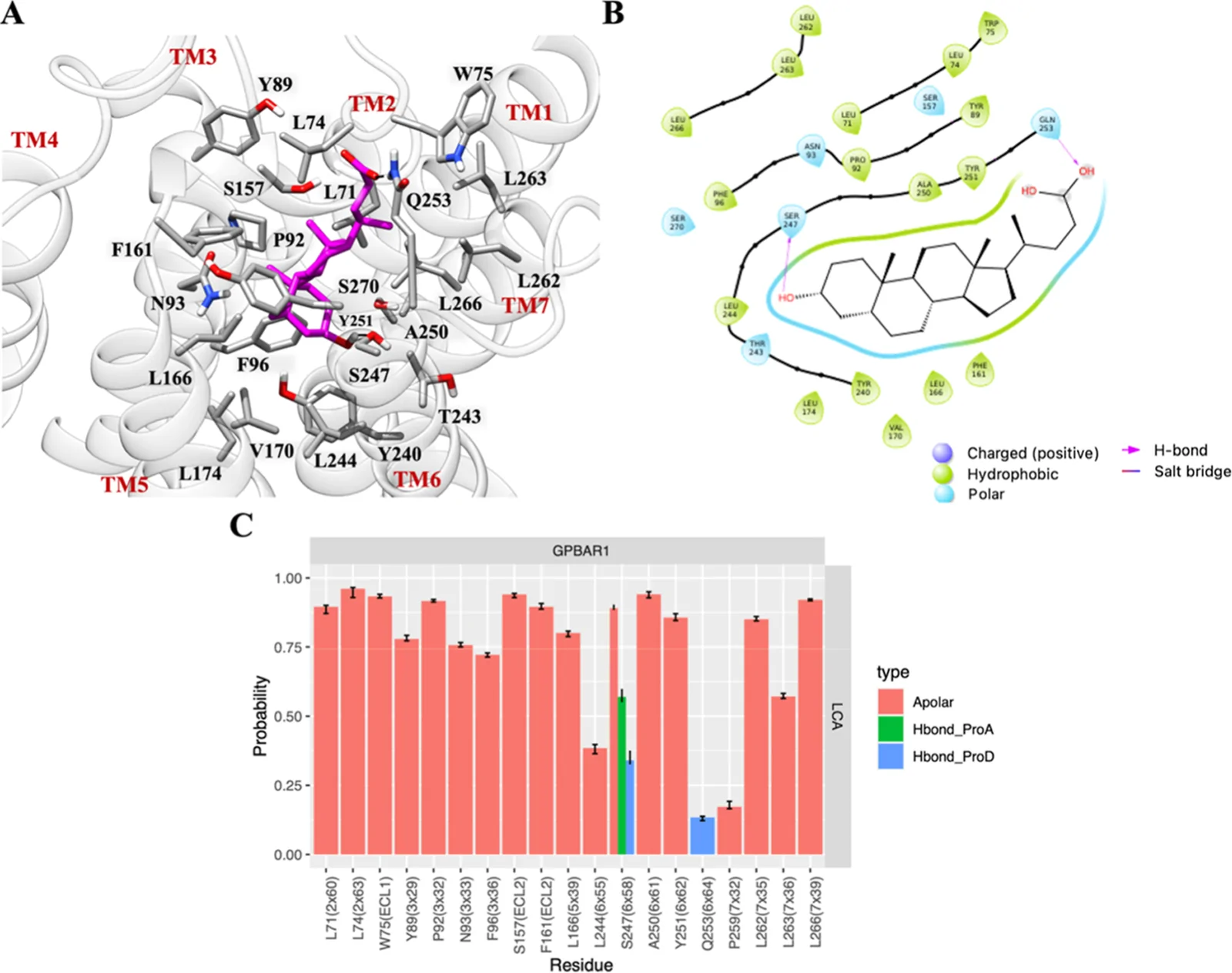
(A) Centroid conformation of the most populated cluster obtained from the three MD replicas of the LCA:GPBAR1 complex. The ligand is represented as magenta sticks, while interacting residues of the receptor are shown in gray and labeled; oxygen and nitrogen atoms are colored red and blue, respectively. The receptor is shown as ribbons with helices labeled. H-bonds are displayed as black dashed lines. (B) LID of the MD-derived centroid of the most populated LCA binding mode in the GPBAR1 receptor. (C) SIFt analyses showing probabilities of ligand–receptor interactions formed during three 500 ns MD simulations of the LCA:GPBAR1 system. Interaction types include apolar carbon–carbon contacts (Apolar, pink), hydrogen bonds with the protein as an acceptor (Hbond_ProA, green), and hydrogen bonds with the protein as a donor (Hbond_ProD, blue). Only interactions with an average probability greater than 10% are displayed.

SIFt analysis confirmed that apolar contacts dominate ligand recognition also in the absence of G protein, with several hydrophobic interactions displaying probabilities above 80–90% during the simulations (Figure 4C). Compared with the LCA:GPBAR1:G_s_ complex, the overall binding mode remains largely conserved.

These observations indicate that G-protein coupling is not required to maintain the overall orthosteric binding mode of LCA. Instead, G_s_ primarily fine-tunes the local organization of the binding pocket, modulating the relative contribution of specific polar interactions while preserving the hydrophobic framework that is responsible for ligand stabilization.

### 3.2 LCA Binding Biases GPBAR1 toward Active-like Conformational Ensembles

To investigate whether LCA binding biases the GPBAR1 conformational ensemble toward active-like states, we performed a comparative analysis of the GPBAR1:G_s_, LCA:GPBAR1:G_s_, roGPBAR1, and LCA:GPBAR1 trajectories, particularly focusing on TM5 and TM6 rearrangements and key activation microswitches. First, we carried out a PCA on the Cα atoms of TM5 and TM6 across the LCA-bound systems.

The first PC captures the largest collective motions involving TM5 and TM6, which are sampled with distinct amplitudes and directions in the LCA:GPBAR1:G_s_ versus GPBAR1:G_s_ systems (Movie S1). In the LCA:GPBAR1:G_s_ system, the conformational landscape spans a broader region of PC space (Figure 5E), with the dominant collective motions involving an outward displacement of TM6 together with coordinated rearrangements of TM5. These motions are associated with the opening of the intracellular side of the receptor (Figure 5A). By contrast, the GPBAR1:G_s_ system explores a more restricted conformational space, populating a compact region of the PC space corresponding to conformations in which TM5 and TM6 remain more inward and closer to inactive-to-intermediate arrangements of the receptor (Figure 5B,F).

**Figure 5:**
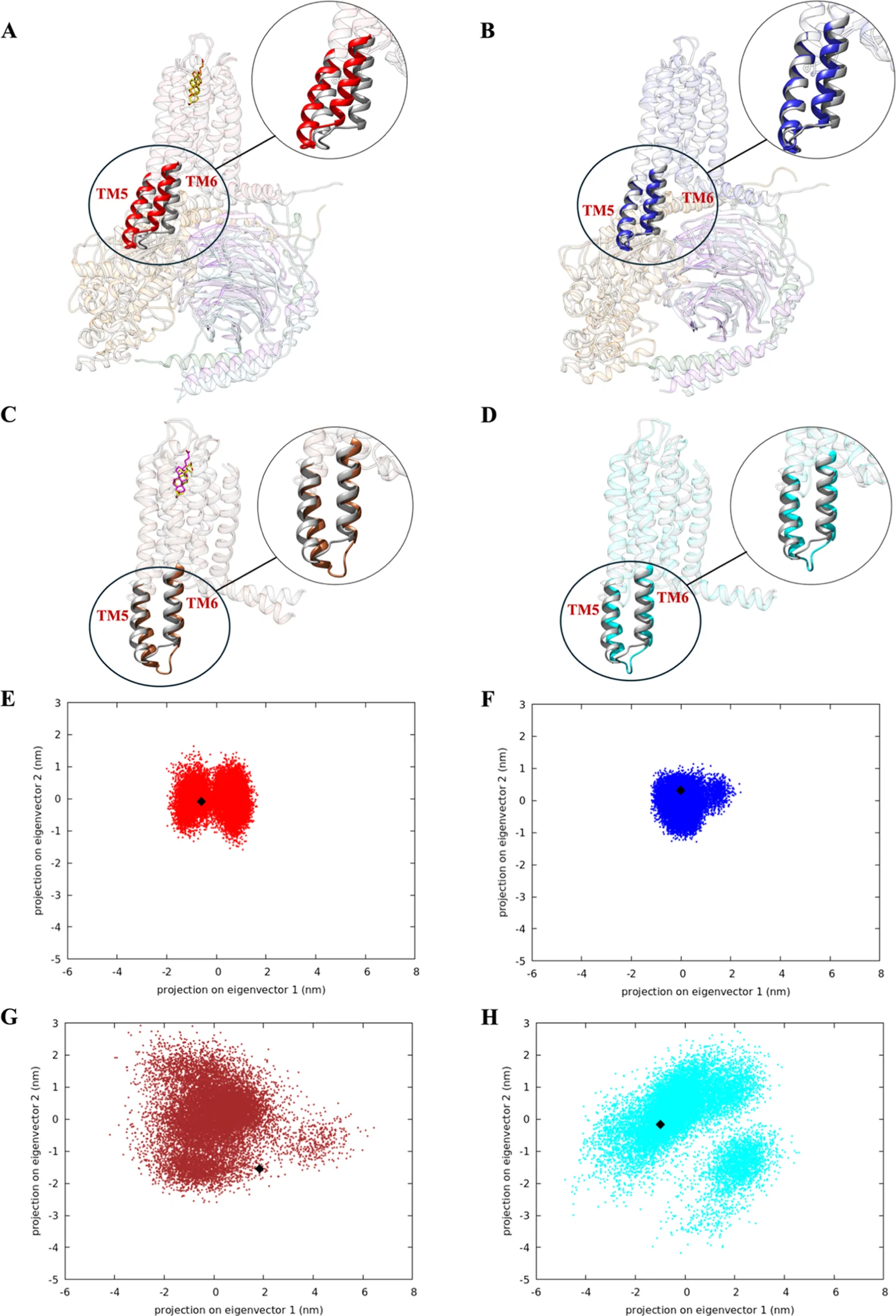
(A–D) Superposition of the starting GPBAR1 state and MD-representative conformations extracted from the most populated clusters for LCA:GPBAR1:G_s_ (A), GPBAR1:G_s_ system (B), LCA:GPBAR1 (C), and roGPBAR1 (D) systems. Initial GPBAR1 structures are shown in gray, while MD-derived conformations are depicted in red (LCA:GPBAR1:G_s_), blue (GPBAR1:G_s_), brown (LCA:GPBAR1), and cyan (roGPBAR1), respectively. Enlarged views highlighting TM5 and TM6 rearrangements are shown in the insets. Two-dimensional (2D) projections of the first two PCs (PC1 and PC2), computed from a combined PCA of the Cα atoms of TM5 and TM6, are shown for the LCA:GPBAR1:G_s_ and GPBAR1:G_s_ (panels E and F), and LCA:GPBAR1 and roGPBAR1 (panels G and H) systems. The positions of the initial MD structures (*t* = 0 ns) are indicated by black rhombuses in panels E–H.

To better characterize how ligand binding and G protein coupling reshape the receptor conformational landscape, we extended the PC analysis to the LCA:GPBAR1 and roGPBAR1 trajectories. In both systems, the receptor notably explores broader and more heterogeneous conformational landscapes compared to the corresponding G protein-bound complexes (Figure 5G,H), indicating that G protein coupling biases the conformational ensemble toward more compact activation-associated states. Notably, roGPBAR1 sampled a larger conformational area with two partially distinct basins (Figure 5H), whereas in LCA:GPBAR1, the distribution was more restricted and asymmetric, with a dominant, broad basin and a secondary tail along PC1 (Figure 5G), consistent with agonist binding biasing the receptor toward a preferred, albeit transiently sampled, active-like region of conformational space. Representative structures extracted from the most populated clusters further support this interpretation, highlighting ligand-dependent rearrangements of TM5 and TM6 that become more pronounced and structurally stabilized upon G_s_ engagement (Figure 5A–D). Consistently, visualization of the dominant PCA motions for the receptor-only systems (Movie S2) reveals a tendency of TM5 and TM6 to undergo inward motions that partially reduce the intracellular opening of the receptor, indicating that, in the absence of G protein, activation-associated conformations are sampled but not persistently maintained.

Consistent with the PCA results, analysis of TM helix rearrangements further supports a ligand-dependent reorganization of the intracellular region of GPBAR1. Across all four simulated systems, the extent and persistence of TM5 and TM6 outward displacement varied systematically as a function of both ligand occupancy and G protein engagement, as evidenced by RMSD and RMSF analyses (Figures S1B–D,F–H,J–L,N–P,R–T and S5–S7). In the LCA:GPBAR1:G_s_ system, TM5 and TM6 undergo a more pronounced and persistent outward displacement that is consistently maintained across all replicas (Figures S1B,F,J,N,R and S5). This observation is further supported by quantitative analysis of diagnostic Cα distances between residues located on TM3 and TM6, identified by Yang et al.,^21^ which reveals an increase in the separation of the two helices in the LCA:GPBAR1:G_s_ system compared to the GPBAR1:G_s_ receptor (Figure 6).

**Figure 6:**
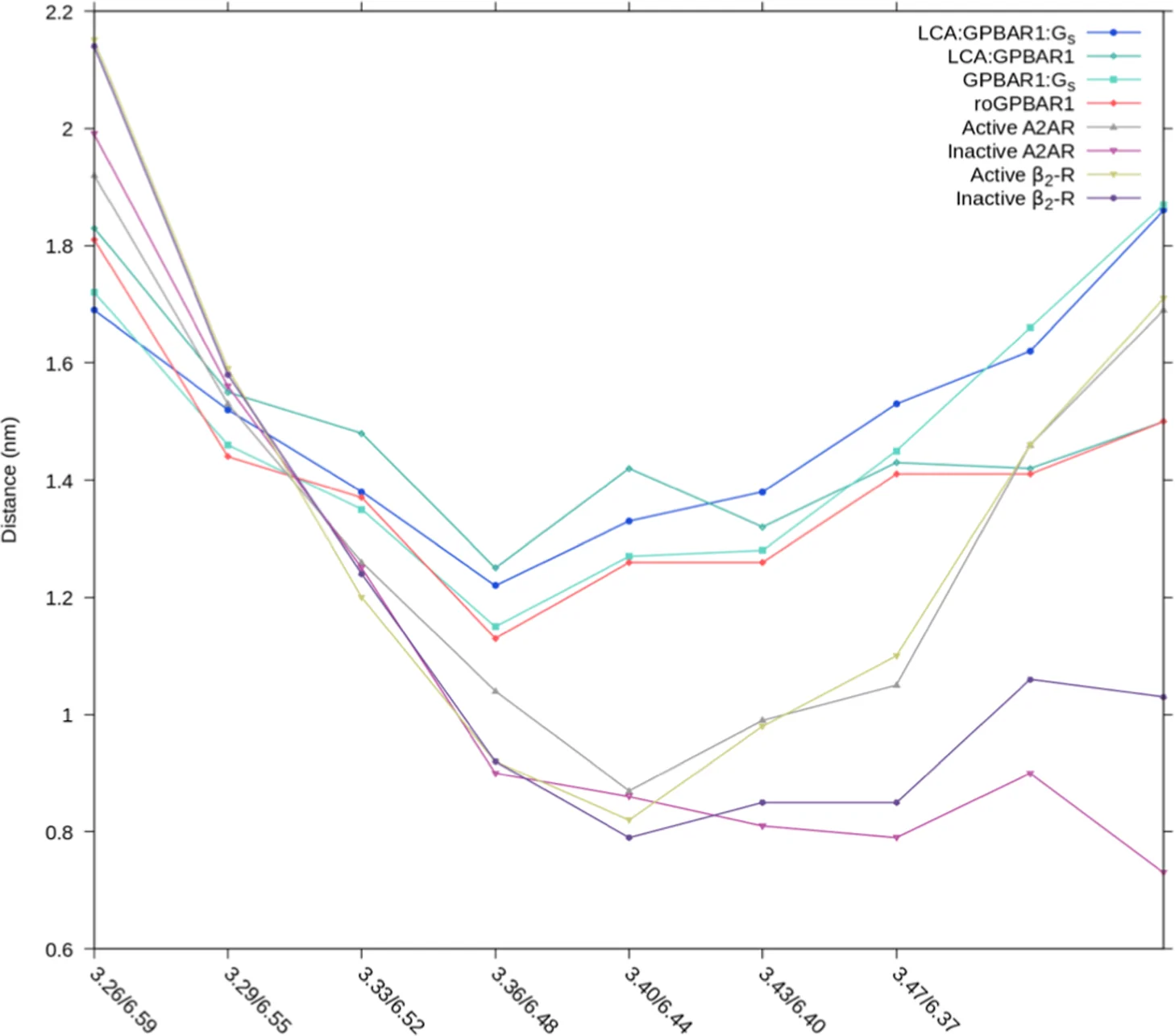
Plot of Cα distances between residues on TM3 and TM6 for the centroids of the most populated cluster obtained from the LCA:GPBAR1:G_s_, GPBAR1:G_s_, LCA:GPBAR1, and roGPBAR1 systems, alongside the active (PDB ID: 6GDG) and inactive (PDB ID: 3VG9) A2AR, and the active (PDB ID: 3S6N) and inactive (PDB ID: 3YNA) β_2_-AR. The *x*-axis shows the Ballesteros–Weinstein residue numbering used to define the TM3-TM6 residue pairs.

Comparison with the G protein-free systems indicates that ligand binding alone is sufficient to reshape the intracellular conformational landscape of GPBAR1 and to promote partial outward rearrangements of TM5 and TM6, as also reflected by the RMSD profiles of these regions (Figure S1C,D,G,H,K,L,O,P,S,T). In agreement with the broader conformational ensembles observed by PCA, RMSF analyses reveal a slightly enhanced structural flexibility in both LCA:GPBAR1 and roGPBAR1 systems than in the corresponding G_s_-bound complexes, particularly within intracellular and loop regions (Figures S6 and S7). Together, these observations indicate that G-protein coupling contributes to constraining and stabilizing activation-associated conformational states. The TM3-TM6 distance profiles further support these findings: LCA promoted a consistent increase in the separation of the two helices both in the presence and in the absence of G_s_, with LCA:GPBAR1:G_s_ displaying the largest distances across all residue pairs, followed by LCA:GPBAR1, and with the two ligand-free systems (GPBAR1:G_s_ and roGPBAR1) showing comparable and systematically lower values (Figure 6). These results indicated that agonist binding is the primary driver of intracellular opening, whereas G protein engagement further stabilizes and organizes the resulting active-like conformational ensemble.

Although the magnitude of TM3-TM6 separation is larger in GPBAR1 than in canonical class A GPCRs, active-state structures of the A2A adenosine receptor (A2AR) (PDB ID: 6GDG) and the β_2_-adrenergic receptor (β_2_-AR) (PDB ID: 3S6N) nonetheless display consistently wider TM3-TM6 distances relative to their corresponding inactive, G protein-free conformations (A2AR, PDB ID: 3VG9; β_2_-AR, PDB ID: 3YNA). These observations are qualitatively consistent with activation-associated TM6 rearrangements previously reported for β_2_-AR and A2AR, while also highlighting receptor-specific features of GPBAR1 conformational dynamics.

By contrast, the GPBAR1:G_s_ simulation exhibited higher stability at the TM bundle level, as reflected by the lower backbone RMSD values for the receptor and for TM5/TM6 relative to the corresponding LCA:GPBAR1:G_s_ system (Figure S1A,E,I,M,Q versus Figure S1B,F,J,N,R). The corresponding roGPBAR1 simulations are reported in Figure S1C,G,K,O,S, while the LCA:GPBAR1 systems are shown in Figure S1D,H,L,P,T. These RMSD profiles indicated that removal of G_s_ increased the conformational freedom of the receptor, consistent with the broader and more heterogeneous conformational landscapes observed in the PCA analysis.

Additional support for ligand-induced stabilization of an active-like conformational ensemble was provided by conformational cluster analysis performed using the GROMOS algorithm.^48^ In the GPBAR1:G_s_ system, the trajectory is distributed across multiple conformational clusters, with the most populated cluster accounting for only 33% of the simulation frames, followed by several additional clusters with lower populations (Table S2). This broad distribution reflects increased conformational heterogeneity in the absence of a ligand, despite the overall stability of the TM bundle. In contrast, the LCA:GPBAR1:G_s_ system predominantly samples two closely related conformational clusters, representing 27% and 26% of the frames, respectively (Table S3). These clusters are structurally similar, with a backbone RMSD of 0.16 nm, indicating that LCA binding biases GPBAR1-G_s_ conformational landscape toward a narrower active-like basin while still allowing fluctuations between closely related conformations. Cluster analysis of the G protein-free systems further revealed a broader conformational distribution compared to the corresponding G_s_-bound complexes. In particular, roGPBAR1 did not exhibit highly populated conformational states, whereas LCA:GPBAR1 sampled two partially populated clusters accounting for 25% and 15% of the trajectory frames (Tables S4 and S5). These results, together with the RMSF profiles observed for the G protein-free systems (Figures S6 and S7), indicate that ligand binding partially restricts the conformational heterogeneity of GPBAR1 even in the absence of G protein, although a complete stabilization of active-like conformational ensembles appears to require G_s_ engagement.

### 3.3 Ligand-Dependent Remodeling of Activation Microswitches and GPBAR1-Gα_s_ Interface

To investigate how the ensemble-level differences observed upon LCA binding translate into local activation features, we analyzed key receptor microswitches and intracellular motifs involved in G-protein coupling in both the LCA:GPBAR1:G_s_ and GPBAR1:G_s_ systems.

Trp^6.48^ is commonly regarded as the toggle switch in class A GPCRs, but previous reports have established that in GPBAR1, this functional role is primarily fulfilled by Tyr240^6.51^ rather than by Trp237^6.48^.^21^ In agreement with this notion, analysis of the residue z-position along the membrane normal revealed that Tyr240^6.51^ occupies a lower position in the LCA:GPBAR1:G_s_ system compared to the GPBAR1:G_s_ receptor (Figure S2A). Notably, an analogous downward displacement was also observed in the G protein-free simulations, where Tyr240^6.51^ adopted lower z-position values in the LCA:GPBAR1 system than in roGPBAR1. Importantly, the z-position distributions of LCA:GPBAR1:G_s_ and LCA:GPBAR1 were nearly superimposable, indicating that the ligand is the primary determinant of this rearrangement and that G-protein coupling contributes marginally to the z-position of Tyr240^6.51^. These observations indicate that agonist binding alone is sufficient to promote activation-associated rearrangements of this microswitch, whereas G-protein coupling mainly contributes to the stabilization of the resulting active-like conformational ensemble. This ligand-dependent downward displacement of Tyr240^6.51^ highlights its central role as the primary sensor of activation in GPBAR1.

A similar trend was observed for Trp237^6.48^ (Figure S2B), which also exhibited lower z-position values in the ligand-bound systems relative to the corresponding ligand-free receptors, both in the presence and in the absence of G protein. However, the magnitude of these changes was smaller than that observed for Tyr240^6.51^, supporting the notion that Trp237^6.48^ participates in downstream conformational rearrangements within TM6 and contributes to the stabilization of the active-like state rather than acting as the principal activation trigger.

GPBAR1 lacks the conserved NPxxY motif found in many class A GPCRs, while the canonical DRY motif is present as an ERY sequence.^21^ To assess the functional relevance of this motif in the context of G protein coupling, we monitored the hydrogen bond established between Glu109^3.49^ and Tyr291 of Gα_s_ in the reference cryo-EM structure throughout the simulations (Figure S3). This interaction is preferentially formed and stabilized in the presence of LCA, whereas in the GPBAR1:G_s_ system it is either transient or absent, indicating that agonist binding promotes productive engagement of the ERY motif with the G protein. In addition, the inactivating ionic lock involving Arg110^3.50^, commonly observed in class A GPCRs, is intrinsically disrupted in GPBAR1 due to the substitution of the conserved counterpart Glu^6.30^ with a threonine. This residue may be capable of engaging in a hydrogen bond with Arg^3.50^ in the inactive state of the receptor, although this possibility would require dedicated investigation.

Consistent with the microswitch analysis and the TM5/TM6 rearrangements identified by PCA, ligand-dependent conformational differences propagated toward the intracellular side of the receptor. In the LCA:GPBAR1:G_s_ system, the coordinated outward motion of TM5 and TM6 promotes an increased opening of the G protein-binding cavity, thereby facilitating interactions with the C-terminal α5 helix of Gα_s_. The enhanced separation between TM5 and TM6 was dynamically correlated with increased flexibility and structural deviations of the α5 helix and the AHD of Gα_s_ (Figure S1U–X), indicating increased conformational heterogeneity at the receptor-G protein interface. Such behavior suggests that LCA binding may allosterically contribute to the early stages of G-protein engagement. In contrast, the Gβ and Gγ subunits remained conformationally stable in both GPBAR1:G_s_ and LCA:GPBAR1:G_s_ simulations, exhibiting only minor positional fluctuations (Figure S1A′–D′).

Representative structures extracted from the most populated clusters further highlight pronounced differences between the GPBAR1:G_s_ and LCA:GPBAR1:G_s_ states at the intracellular G protein-coupling interface (Figure 5A,B). The GPBAR1:G_s_ system, which represents the basal, ligand-independent conformational ensemble of GPBAR1, exhibited higher structural stability, as reflected by the RMSD and RMSF profiles (Figures S1, S4 and S5). This observation is consistent with previous reports describing a constitutive basal activity of GPBAR1 in the absence of bound ligands.^66^ In this state, the α5 helix remains stably inserted into the receptor cavity, supported by a hydrophobic cleft formed by residues from TM3, TM5, and TM6 (Figures 5B and S4). In contrast, in the LCA:GPBAR1:G_s_ system (Figures 5A and S5), the increased flexibility of TM5 and TM6 occasionally facilitates the unraveling and partial disengagement of the α5 helix from the receptor core. To quantitatively characterize this behavior, the radius of gyration (*R*_g_) of the α5 helix was calculated using the GROMACS analysis tool. As shown in Figure S8, the LCA:GPBAR1:G_s_ simulations exhibit slightly higher *R*_g_ values compared to the GPBAR1:G_s_ simulations, indicative of an increased conformational spread of the α5 helix in the presence of LCA. This behavior is consistent with rearrangements reported for early stages of G protein activation in agonist-bound GPCR complexes.^67,68^

### 3.4 Ligand Binding Enhances Specific Allosteric Communication Pathways to the G Protein Interface

To better discriminate the structural and dynamic differences between the LCA:GPBAR1:G_s_ and GPBAR1:G_s_ states of the human bile acid receptor GPBAR1, we conducted a network-based analysis of allosteric communication pathways using the MDpath tool.^54^ This approach infers residue-level communication routes by quantifying correlated atomic fluctuations along MD trajectories and modeling them as probabilistic paths within a graph representation of the receptor (see the Methods section for details).

This analysis identified the most probable communication routes connecting the TM helices and the intracellular region involved in G-protein coupling. In both systems, the detected pathways are largely confined to the helical core of the receptor; however, clear differences emerged between the GPBAR1:G_s_ and LCA:GPBAR1:G_s_ states. In the GPBAR1:G_s_ system, the residues most involved in signal propagation are primarily located in TM5, whereas in the LCA:GPBAR1:G_s_ system, they mainly belong to TM6, indicating a distinct redistribution of allosteric communication pathways between the two conformational ensembles (Figure S9).

In the absence of ligand, dominant communication paths mostly involve the central region of the helical bundle, particularly those involving TM3 and TM6, but do not efficiently connect the orthosteric pocket to the intracellular G-protein coupling interface (Figures 7A and S9A,B). These compact intrahelical networks are consistent with a more constrained intracellular region and with the partially relaxed active-like ensembles sampled in the GPBAR1:G_s_ simulations, in which microswitch rearrangements remain dynamically heterogeneous and do not converge toward a single, fully stabilized intracellular configuration associated with productive G-protein engagement.^23,69^

**Figure 7:**
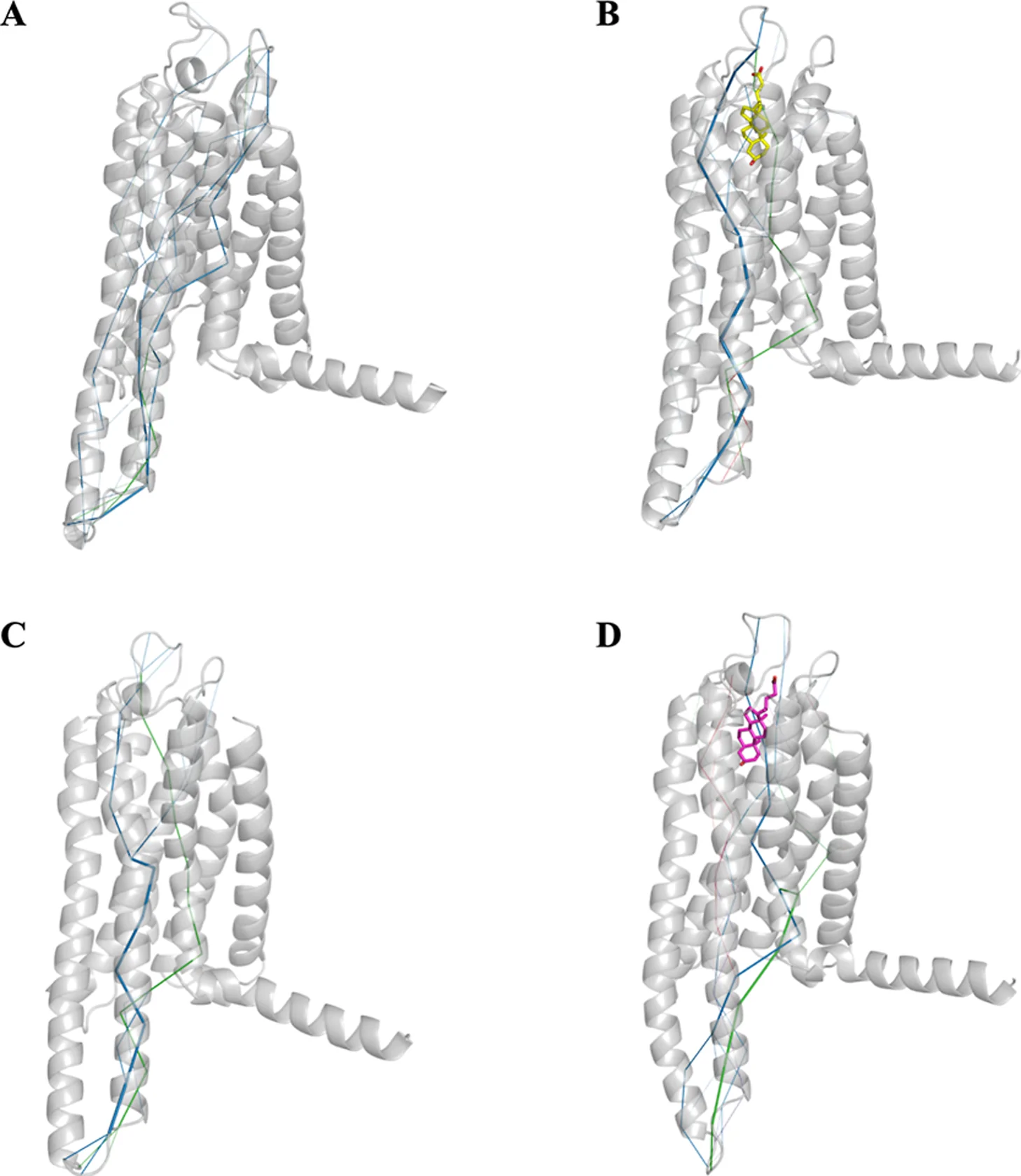
Comparison of allosteric communication pathways in GPBAR1 obtained from the MDpath analysis. Panels A and B correspond to the GPBAR1:G_s_ and LCA:GPBAR1:G_s_ systems, respectively, whereas panels C and D correspond to the roGPBAR1 and LCA:GPBAR1 systems. Communication paths are mapped onto backbone Cα atoms, providing an intuitive representation of the significance of the residue-level allosteric network. Path colors correspond to the cluster assignments, enabling the different communication channels to be distinguished at a glance.

In contrast, LCA binding induces a redistribution of the communication routes toward TM6, reaching the intracellular region. This behavior correlates with the wider TM5-TM6 separation relative to TM3 discussed above and suggests enhanced dynamic coupling between the orthosteric pocket and the G protein coupling interface (Figures 7B and S9A,C). In this context, ligand-induced stabilization of microswitches in the active conformation and the concerted TM5-TM6 rearrangements provide a structural framework for extended allosteric communication toward the G protein binding interface.

A qualitatively consistent pattern was observed in the G-protein-free systems, confirming that ligand-dependent remodeling of the allosteric network occurs independently of G-protein coupling. In the roGPBAR1 system, communication pathways remained fragmented and largely confined within the core of the TM bundle, with limited propagation toward the intracellular region (Figures 7C and S10A,B). The residues contributing most strongly to the network were distributed across TM3, TM5, and TM6, consistent with the broad conformational heterogeneity observed in PCA analysis.

Conversely, the LCA:GPBAR1 system displayed a more organized communication architecture (Figures 7D and S10A,C). Upon agonist binding, the dominant pathways became preferentially redistributed toward TM6 and extended toward the intracellular receptor surface despite the absence of the G protein. This behavior indicates that LCA binding is sufficient to establish long-range dynamic coupling between the orthosteric pocket and intracellular regions associated with signaling. However, compared with the LCA:GPBAR1:G_s_ complex, these communication routes appear less focused and less extensively connected, suggesting that G protein engagement further stabilizes and reinforces the ligand-induced allosteric network.

Visual inspection of representative structures confirmed these findings: in the LCA:GPBAR1:G_s_ state, GPBAR1 (Figures 7B and S9) exhibited a pronounced outward movement of TM6 and a slight rotation compared to the GPBAR1:G_s_ receptor (Figures 7A and S9), resulting in a more open intracellular cavity. Consistently, the dominant allosteric paths in the LCA:GPBAR1:G_s_ are aligned with this movement, running from the orthosteric site toward the cytoplasmic region along TM3-TM5-TM6. Notably, the radius of gyration profiles of TM6 remained largely stable throughout the simulations, both in the GPBAR1:G_s_ and LCA:GPBAR1:G_s_ complexes (Figure S11A,B) and in the corresponding G-protein-free systems (Figure S11C,D). This indicates that the observed activation-associated rearrangements primarily involve changes in the positioning and orientation of TM6 rather than substantial alterations to its overall helical compactness.

Altogether, these results highlight how ligand binding reshapes the internal dynamic network of GPBAR1, promoting the development of extended allosteric pathways that connect the extracellular and intracellular domains, eventually facilitating G protein engagement.

## 4 Discussion

In this work, we present an integrative computational strategy aimed at exploring how LCA modulates the conformational landscape of GPBAR1 and its coupling with the G_s_ protein. The available cryo-EM structures of the GPBAR1-G_s_ complex provide important structural details of the receptor/effector binary complex,^21,70,71^ but these structures lack several highly flexible segments, including the terminal regions of the receptor and G protein, as well as the AHD of Gα_s_, thereby limiting our understanding of the functional dynamics that couple ligand binding, receptor activation, and G protein engagement. To address these limitations, we reconstructed the full receptor-G protein complex and embedded it in a membrane environment, enabling microsecond-scale MD simulations. By comparing four distinct systems, the LCA-bound and ligand-free GPBAR1 receptors, both in the presence and in the absence of G_s_, we investigated how binding of the endogenous agonist LCA influences local interactions within the orthosteric pocket and how ligand binding and G protein coupling contribute, respectively, to the broader conformational features associated with receptor activation.

The ligand binding mode obtained from docking (Figure 2) and subsequently refined using MD simulations (Figure 3) highlighted how the endogenous bile acid leveraged both hydrophobic packing and specific polar contacts within the TM cavity. Importantly, these interactions are dynamic: throughout the MD simulations, the LCA hydroxyl group alternates between forming hydrogen bonds with Tyr240^6.51^ and Ser247^6.58^, while the carboxylate transiently establishes ionic contacts with Arg254^ECL3^. This behavior was captured in the centroid pose derived from clustering (Figures 3 and S1E). Such interaction patterns suggest that LCA accommodates intrinsic receptor flexibility instead of imposing a strictly rigid geometry, thereby modulating the receptor’s energy landscape rather than locking it into a unique conformation. Although no experimental structure of a GPBAR1-LCA complex is currently available, the present simulations provide an atomistic model that may help rationalize the agonistic potency of this endogenous bile acid. In this context, the involvement of Ser247^6.58^ and Arg254^ECL3^ may contribute both to ligand stabilization and to the modulation of ECL dynamics, which are increasingly recognized as integral components of GPCR activation networks.^65,72^ Notably, G protein-free simulations reveal that the overall orthosteric binding mode of LCA is largely preserved even in the absence of G_s_ (Figure 4). Indeed, the steroidal scaffold remains stabilized by the same hydrophobic network observed in the LCA:GPBAR1:G_s_ complex, whereas only minor differences are detected in the organization of polar interactions involving residues of TM6 and ECL3. These observations indicate that agonist recognition is primarily governed by local receptor–ligand interactions within the orthosteric cavity, while G_s_ coupling mainly contributes to fine-tuning the organization of the polar interaction network surrounding the bound ligand.

Beyond the binding pocket, LCA engagement is associated with a redistribution of intramolecular strain and with changes in the behavior of activation-related microswitches. The observed shift in the z-position of Tyr^6.51^ (Figure S2A) and the outward displacement of TM6 are consistent with conformational features commonly associated with class A GPCR activation. These trends are in qualitative agreement with experimental observations reported by Yang et al. and D’Amore et al.,^21,73^ who described coordinated microswitch rearrangements and TM6 outward motion upon agonist binding. They are also consistent with previous studies showing that agonists modulate intracellular TM5-TM7 interaction networks through conformational ensemble redistribution rather than through discrete structural switches.^74^ In the case of GPBAR1, these changes appear particularly sensitive to the positioning of the ligand core, supporting the idea that steroidal agonists act as conformational “wedges” that facilitate the outward displacement of TM6. Notably, comparable Tyr240^6.51^ and Trp237^6.48^ rearrangements are observed in LCA:GPBAR1 relative to roGPBAR1, indicating that agonist binding alone is sufficient to promote activation-associated microswitch transitions. G protein coupling, therefore, appears to act primarily by stabilizing these ligand-induced conformational states rather than by initiating them (Figure S2). This interpretation is further supported by the RMSD and RMSF analyses (Figures S1, S4, and S5), which shows increased mobility of the intracellular tips of TM5 and TM6 in the LCA:GPBAR1:G_s_ system, indicative of a redistribution of flexibility from the extracellular pocket toward the intracellular interface.

The consequences of these reorganizations are clearly visible when comparing the conformational ensembles across the four simulated systems. Clustering analysis indicates that the receptor in the LCA:GPBAR1:G_s_ complex explores a narrower subset of conformations, whereas the GPBAR1:G_s_ system populates a more heterogeneous ensemble. PCA further reinforces this interpretation: the dominant global motion captures the outward displacement of TM6, which is prominently sampled in the LCA:GPBAR1:G_s_ simulations (Figure 5E) but remains limited in the GPBAR1:G_s_ state (Figure 5F). Notably, while the receptor in LCA:GPBAR1:G_s_ explores multiple active-like geometries, these conformations share a conserved intracellular TM6 displacement, consistent with the idea that ligand binding biases the conformational ensemble toward activation-competent states rather than stabilizing a single, unique active structure. Comparison with the G-protein-free systems further helps disentangle ligand-driven and G-protein-stabilized conformational effects. In the absence of G_s_, both the apo and LCA-bound receptor systems sample broader conformational ensembles than the corresponding G_s_-bound systems (Figure 5G,H). Notably, the receptor-only systems displayed distinct conformational distributions. roGPBAR1 sampled a larger conformational space characterized by two partially distinct basins (Figure 5H), indicating increased structural heterogeneity in the absence of both the ligand and G protein. In contrast, the LCA:GPBAR1 system explored a more restricted and asymmetric conformational landscape, consisting of a dominant broad basin and a secondary tail along PC1 (Figure 5G). These observations suggest that agonist binding alone is sufficient to bias GPBAR1 toward preferred activation-associated regions of conformational space, although such states remain less persistently stabilized than in the corresponding G_s_-bound complex. Nevertheless, LCA retains the ability to promote TM5/TM6 rearrangements and intracellular opening, suggesting that agonist binding alone is sufficient to initiate activation-associated conformational changes. These observations support a model in which ligand binding drives the early stages of receptor activation, whereas G protein coupling contributes primarily to the stabilization and functional organization of active-like conformational ensembles. Our results are also consistent with recent findings on the A2A adenosine receptor, where agonist binding alone was shown to populate metastable active-like conformations, whereas stabilization of the fully active state required G-protein engagement.^73,75^ These observations support a conserved class A GPCR activation mechanism in which ligand binding initiates activation-associated conformational changes, while transducer coupling reinforces and stabilizes the resulting active-state ensemble. Consistent with this interpretation, visualization of the dominant PCA motions revealed a tendency of TM5 and TM6 to undergo partial inward movements in the G protein-free systems, suggesting that activation-associated conformations are sampled but not persistently maintained in the absence of G_s_ coupling.

Importantly, the present findings should be interpreted within the broader framework of established GPCR ensemble theory, according to which ligand efficacy arises from the selective stabilization of pre-existing receptor conformations rather than from induction of a single discrete active state.^76–78^ Within this context, GPBAR1 remains substantially less characterized than canonical class A GPCRs, despite its growing pharmacological relevance in metabolic and inflammatory disorders. The present study, therefore, provides a receptor-specific characterization of bile acid-dependent conformational dynamics, highlighting how LCA modulates TM5/TM6 rearrangements, microswitch organization, G protein engagement, and allosteric communication pathways. In addition, reconstruction of the complete Gα_s_ AHD enabled the exploration of activation-associated conformational features that are not accessible from currently available GPBAR1 cryo-EM structures.

The present results obtained for LCA should be interpreted within a broader ligand-dependent framework. Different bile acids, characterized by variations in hydroxylation patterns and side-chain flexibility, are expected to differentially modulate GPBAR1 conformational dynamics, including TM5/TM6 rearrangements, activation-related microswitches, and organization of allosteric communication pathways. In this context, the LCA:GPBAR1:G_s_ system represents one point within a wider conformational landscape, and the present study provides a foundation for future comparative investigations aimed at linking ligand-specific dynamics to distinct physiological outcomes.

Importantly, GPBAR1 has been reported to display ligand-independent (“basal/constitutive”) activity in cellular systems.^66^ In line with this, our GPBAR1:G_s_ model retains partially active intracellular features that can rationalize basal signaling: even without ligand, the receptor intermittently adopts conformations compatible with residual G-protein engagement, thereby providing a structural basis for basal activity.

Additional insight emerges by examining the dynamics of the G protein, particularly the C-terminal α5 helix of Gα_s_. In the LCA:GPBAR1:G_s_ simulations (Figures S3–S5), the α5 helix exhibited enhanced fluctuations and occasional reductions in its depth of insertion into the intracellular cavity. These changes appear to correlate with the ligand-induced rearrangements of TM5 and TM6 and suggest a dynamic coupling between receptor activation and G protein conformational variability. Similar coupling between ligand-dependent receptor conformations and Gα_s_ α5 helix dynamics has previously been described in canonical GPCR systems, supporting the idea that these motions represent conserved features of ternary complex activation.^79^

Transient partial unraveling of α5 and rearrangements are observed within the nucleotide-binding site, particularly at the Ras-AHD interdomain region, which has been associated in previous studies with early stages of G protein activation.^67,68^ While processes such as GDP destabilization and nucleotide exchange cannot be directly resolved on the time scale of the present simulations, the observed conformational trends were compatible with initial priming events that precede these steps and provide a structural context for interpreting how receptor activation may influence G protein readiness.

The network-based analysis of allosteric communication pathways further integrates these observations and provides a dynamic framework to interpret the respective contributions of agonist binding and G-protein coupling to GPBAR1 activation. In the absence of a ligand, communication pathways remain confined to the core of the TM bundle, particularly to the TM3-TM6 region, and do not effectively connect the orthosteric pocket to the intracellular interface. This suggests that GPBAR1:G_s_ lacks an efficient conduit for transmitting structural changes from the orthosteric to the G-protein-binding site. Upon LCA binding, the dominant pathways connect the orthosteric binding site to intracellular regions through TM5-TM6. The concentration of allosteric flow along TM6 in the LCA:GPBAR1:G_s_ state mirrors the structural expansion captured by PCA, highlighting the central role of this helix as a conduit for transmitting ligand-induced rearrangements. These features are consistent with experimentally described microswitch conformations and suggest that the MD simulations capture not only ligand-induced local changes but also global structural propagation that is critical for G protein activation. Nevertheless, important differences emerged between the two ligand-bound systems. In the absence of G protein, the communication network observed in the LCA:GPBAR1 receptor appeared more diffuse and less focused, indicating that although agonist binding promotes activation-associated signal propagation, the resulting pathways remain dynamically heterogeneous. In contrast, in the LCA:GPBAR1:G_s_ complex, communication routes converged more consistently toward the intracellular receptor surface and the G protein-coupling region, generating a more coherent and organized allosteric architecture. The preferential concentration of communication flow along TM6 mirrors the structural expansion captured by PCA and highlights the central role of this helix as a conduit for transmitting ligand-induced conformational changes across the receptor.

Taken together, these results support a hierarchical mechanism of GPBAR1 activation in which agonist binding initiates the formation of long-range allosteric communication pathways, whereas G protein coupling reinforces and stabilizes these routes into signaling-competent networks. This interpretation is fully consistent with the conformational ensemble analysis, microswitch dynamics, and receptor-G protein interface rearrangements described above, which collectively indicate that LCA acts as the primary initiator of activation-associated conformational changes, while G_s_ engagement consolidates the resulting active-like conformational states.

## 5 Conclusion

In this study, we employed an integrative computational strategy combining structural modeling, molecular docking, microsecond-scale MD simulations, and network analysis to investigate the molecular and dynamic determinants of GPBAR1 activation by its endogenous agonist, LCA. By reconstructing a full-length GPBAR1-G_s_ assembly, we extended the information provided by available cryo-EM structures and explored receptor behavior beyond static conformations, capturing ligand-dependent modulation of its conformational landscape.

The simulations demonstrate that LCA binding stabilizes GPBAR1 toward a dynamically restricted ensemble of active-like conformations rather than a single well-defined active state. This ensemble is characterized by outward movements of TM5 and TM6 and by coordinated rearrangements of activation-related microswitches, including the GPBAR1-specific Tyr240^6.51^ toggle switch. Comparative simulations performed in the absence of G protein further revealed that agonist binding alone is sufficient to promote many of these activation-associated rearrangements, including TM5/TM6 reorganization and microswitch transitions, whereas G_s_ coupling contributes primarily to the stabilization of more organized active-like conformational ensembles.

At the orthosteric site, the dynamic binding mode of LCA highlights the contribution of transient polar contacts and a conserved hydrophobic network involving TM3, TM5, and TM6 along with ECLs. These features, not readily accessible from static experimental structures, underscore the importance of ligand-induced dynamic stabilization in determining agonist efficacy, provide additional insight into the molecular basis of ligand recognition and potency in GPBAR1, and may inform future structure–activity relationship studies.

Consistent with this view, analysis of allosteric communication pathways suggests that ligand binding reorganizes internal signaling routes, enhancing connectivity between the orthosteric pocket and the intracellular coupling interface. Altogether, these findings support a model in which GPBAR1 activation emerges from distributed, ligand-dependent conformational changes across the receptor rather than from a single structural transition. The G-protein-free simulations further suggest that agonist binding initiates activation-associated structural rearrangements, while G-protein engagement stabilizes and functionally organizes these conformational states into signaling-competent ensembles.

Although the present MD simulations capture dominant transitions associated with GPBAR1 activation, a complete mapping of the receptor’s metastable landscape will require enhanced-sampling methodologies and AI-assisted approaches, which are increasingly successful at resolving transient or experimentally inaccessible GPCR states that may prove crucial for ligand discovery and functional selectivity.^73,80^

In conclusion, this study provides a detailed receptor-specific characterization of GPBAR1 activation by the endogenous bile acid LCA, offering mechanistic insights into a GPCR whose activation dynamics remain considerably less explored than those of canonical class A receptors. By integrating comparative modeling, microsecond-scale simulations, and network-based analyses, the present work contributes to a more comprehensive understanding of bile-acid-dependent receptor activation and provides a structural background for future studies that may guide the rational design of next-generation ligands aimed at treating metabolic and inflammatory disorders.

## Supplementary Material







## Data Availability

The data that support the findings of this study are openly available in Zenodo at www.zenodo.org, reference DOIs: 10.5281/zenodo.17883284 (v1), 10.5281/zenodo.20341627 (v2). All other data are available upon request.

## Notes

Supporting animation regarding the PCA of LCA:GPBAR1:G_s_, GPBAR1:G_s_, LCA:GPBAR1, and roGPBAR1 systems (MPG)
PCA of GPBAR1 systems (MPG)
Average RMSD plots of the three 500 ns-long MD replicas; violin plot showing the vertical displacement (z-position) of the toggle switches Tyr^6.51^ and Trp^6.48^; time evolution of the hydrogen-bond distance between Glu109^3.49^ of the ERY motif in GPBAR1 and Tyr291 of the Gα_s_ α5 helix over three independent MD simulation replicas; RMSF analysis of the GPBAR1:G_s_ system computed over three independent 500 ns MD replicas; RMSF analysis of the LCA:GPBAR1:G_s_ system computed over three independent 500 ns MD replicas; RMSF analysis of the LCA:GPBAR1 system computed over three independent 500 ns MD replicas; RMSF analysis of the roGPBAR1 system computed over three independent 500 ns MD replicas; radius of gyration (*R*_g_) computed on the Gα_s_ α5-helix backbone over three independent replicas (500 ns each); allosteric communication pathways in GPBAR1 obtained from MDpath analysis; allosteric communication pathways in GPBAR1 in systems without G_s_ obtained from MDpath analysis; radius of gyration (*R*_g_) computed on the GPBAR1 receptor TM6 backbone over three independent replicas (500 ns each); structural comparison of available GPBAR1 cryo-EM structures considered for template selection; cluster analysis of 1500 ns MDs of GPBAR1:G_s_ system; cluster analysis of 1500 ns MDs of LCA:GPBAR1:G_s_ system; cluster analysis of 1500 ns MDs of roGPBAR1 system and; cluster analysis of 1500 ns MDs of LCA:GPBAR1 system (PDF)

## Abbreviations

GPBAR1: G protein-coupled bile acid receptor 1
TGR5: Takeda G protein-coupled receptor 5
AHD: α-helical domain
LCA: lithocholic acid
SAR: structure–activity relationship
TM: transmembrane helix
ECL: extracellular loop
MD: molecular dynamics
PCA: principal component analysis
RMSD: root mean square deviation
RMSF: root mean square fluctuation
*R*_g_: radius of gyration
NMI: normalized mutual information
SIFt: structural interaction fingerprint
GUI: graphical user interface
PRCG: Polak–Ribiere conjugate gradient
OPLS: optimized potentials for liquid simulations
CGenFF: CHARMM general force field
DOPE: discrete optimized protein energy
cryo-EM: cryo-electron microscopy
PDB: Protein Data Bank
PBC: periodic boundary conditions
POPC: 1-palmitoyl-2-oleoyl-*sn*-glycero-3-phosphocholine
CHL: cholesterol
GlcNAc: N-acetylglucosamine
cAMP: cyclic adenosine monophosphate
PKA: protein kinase A
GLP-1: glucagon-like peptide-1
IBD: inflammatory bowel disease
