# Supplementary material for: From Structure to Dynamics: Activation Mechanism of the G Protein-Coupled Bile Acid Receptor 1‑Gs Complex
Source: ACS Omega. 2026 Aug 13;11(33):49402–19. doi: 10.1021/acsomega.6c02725 (PMC13520505; doi:10.1021/acsomega.6c02725)
Supplement: Supplementary file 1 [file ao6c02725_si_003.pdf]

Supporting Information

FROM STRUCTURE TO DYNAMICS: ACTIVATION MECHANISM OF THE G PROTEIN-COUPLED BILE ACID RECEPTOR 1-G_s COMPLEX

Bianca Fiorillo,[1,] Federica Moraca,[1] Francesco Saverio Di Leva,[1] Valentina Sepe,[1] Stefano Fiorucci,[2] Vittorio Limongelli,[3] Angela Zampella,[1] Bruno Catalanotti[1,*]*

[1] Department of Pharmacy, University of Naples "Federico II", Via D. Montesano, 49, 80131 Naples, Italy

[2] Department of Medicine and Surgery, University of Perugia, Piazza L. Severi, 06132 Perugia, Italy

[3] Faculty of Biomedical Sciences, Euler Institute, Università della Svizzera italiana (USI), via G. Buffi, 13, CH-6900 Lugano, Switzerland

*To whom correspondence should be addressed.
Bianca Fiorillo Phone: (+39) 081678551 E-mail: bianca.fiorillo@unina.it
Bruno Catalanotti Phone: (+39) 081678551 E-mail: brucatal@unina.it

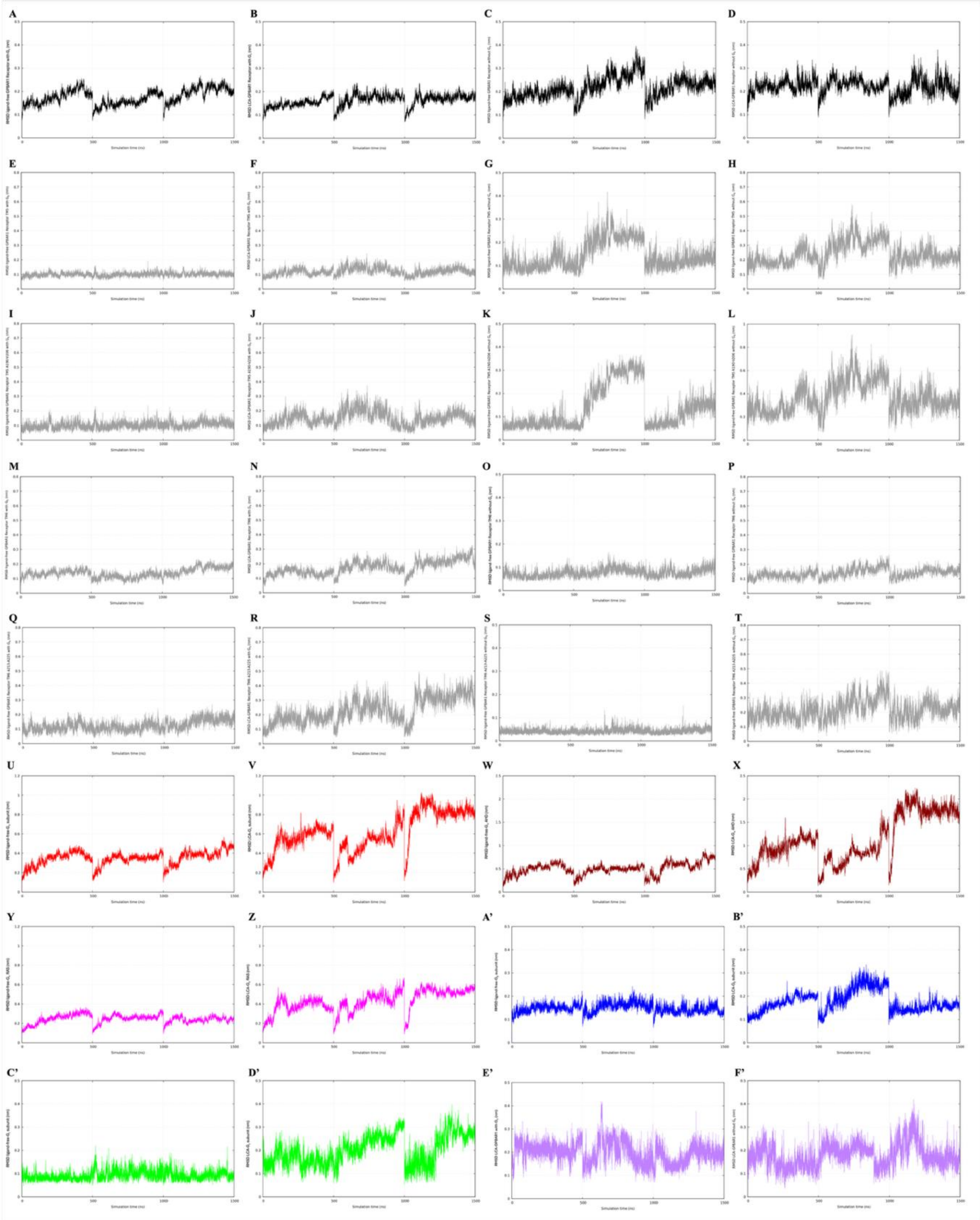

Figure S1. RMSD plots of the three 500 ns-long concatenated MD replicas calculated for: A-D) the GPBAR1 backbone in GPBAR1:G$_s$, LCA:GPBAR1:G$_s$, roGPBAR1 and LCA:GPBAR1, respectively; E-H) the TM5 backbone in GPBAR1:G$_s$, LCA:GPBAR1:G$_s$, roGPBAR1 and LCA:GPBAR1, respectively; I-L) the intracellular portion of TM5 in GPBAR1:G$_s$, LCA:GPBAR1:G$_s$, roGPBAR1 and LCA:GPBAR1, respectively; M-P) the

TM6 backbone in GPBAR1:G_s, LCA:GPBAR1:G_s, roGPBAR1 and LCA:GPBAR1, respectively; Q-T) the intracellular portion of TM6 in GPBAR1:G_s, LCA:GPBAR1:G_s, roGPBAR1 and LCA:GPBAR1, respectively; U-V) the Gα subunit backbone in the GPBAR1:G_s and LCA:GPBAR1:G_s systems, respectively; W-X) the $G\alpha_s$ α-helical domain (AHD) backbone in the GPBAR1:G_s and LCA:GPBAR1:G_s systems, respectively; Y-Z) the $G\alpha_s$ RAS domain backbone in the GPBAR1:G_s and LCA:GPBAR1:G_s systems, respectively; A'-B') the Gβ subunit backbone in the GPBAR1:G_s and LCA:GPBAR1:G_s systems, respectively; C'-D') the Gγ subunit backbone in the GPBAR1:G_s and LCA:GPBAR1:G_s systems, respectively; E'-F') the ligand heavy atoms of LCA in the LCA:GPBAR1:G_s and in the LCA:GPBAR1 systems, respectively.

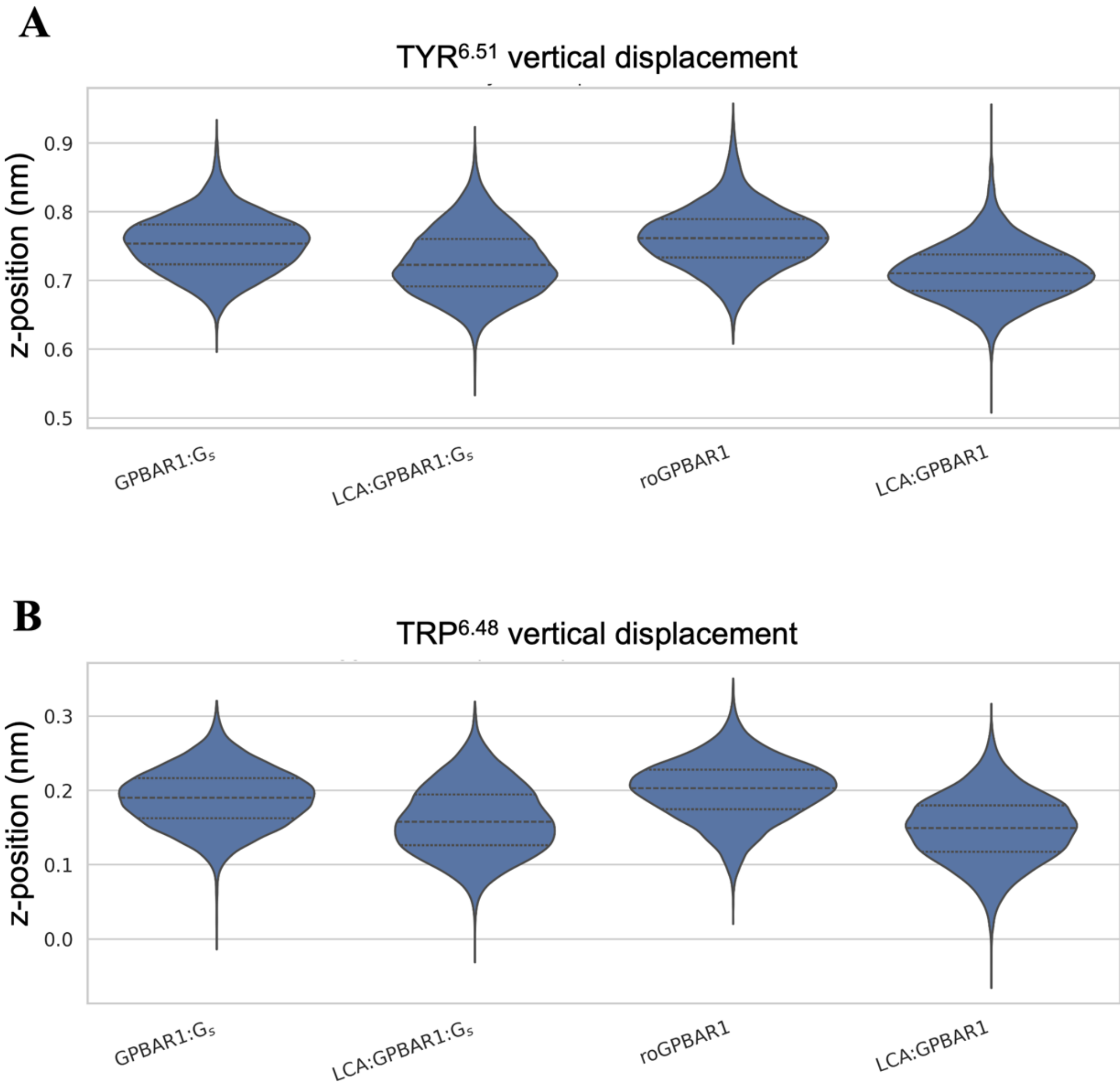

Figure S2. Violin plot showing the vertical displacement (z-position) of A) the toggle switches Tyr$^{6.51}$ and B) Trp$^{6.48}$, calculated as the center-of-mass position of each residue in the GPBAR1:G$_s$, LCA:GPBAR1:G$_s$, roGPBAR1, and LCA:GPBAR1 systems throughout the MD simulations. Prior to the analysis, all trajectories were structurally aligned on the GPBAR1 transmembrane helices excluding TM6, in order to isolate local activation-associated rearrangements of the TM6 microswitch region. Positional values are reported in nanometers (nm).

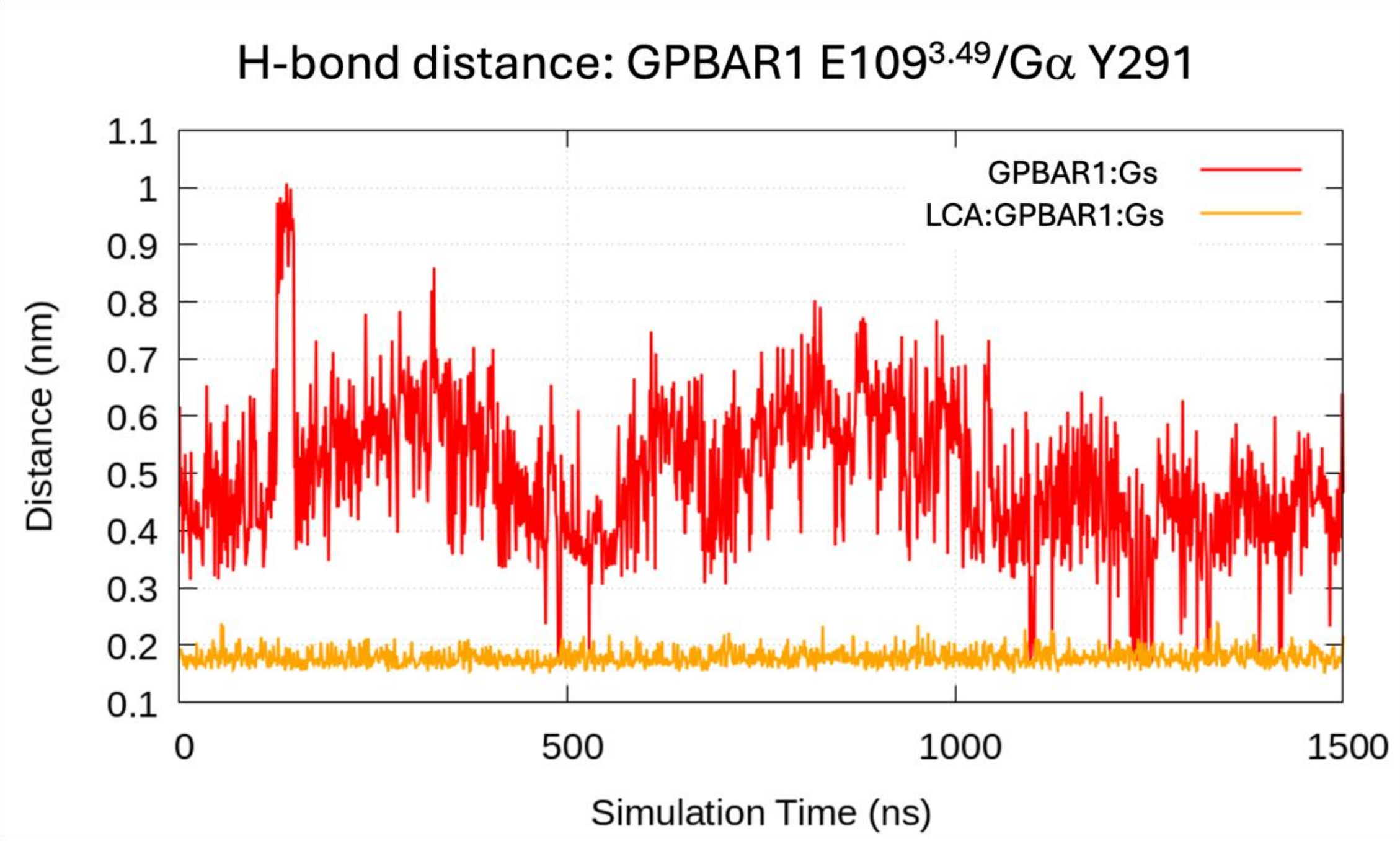

Figure S3. Time evolution of the hydrogen-bond distance between Glu109$^{3.49}$ of the ERY motif in GPBAR1 and Tyr291 of the Gα$_s$ α5 helix over three independent MD simulation replicas. Distances are shown for both the GPBAR1:G$_s$ and LCA:GPBAR1:G$_s$ systems, highlighting that the interaction is maintained predominantly in the LCA:GPBAR1:G$_s$ complex, indicative of ligand-stabilized coupling between the receptor and G protein.

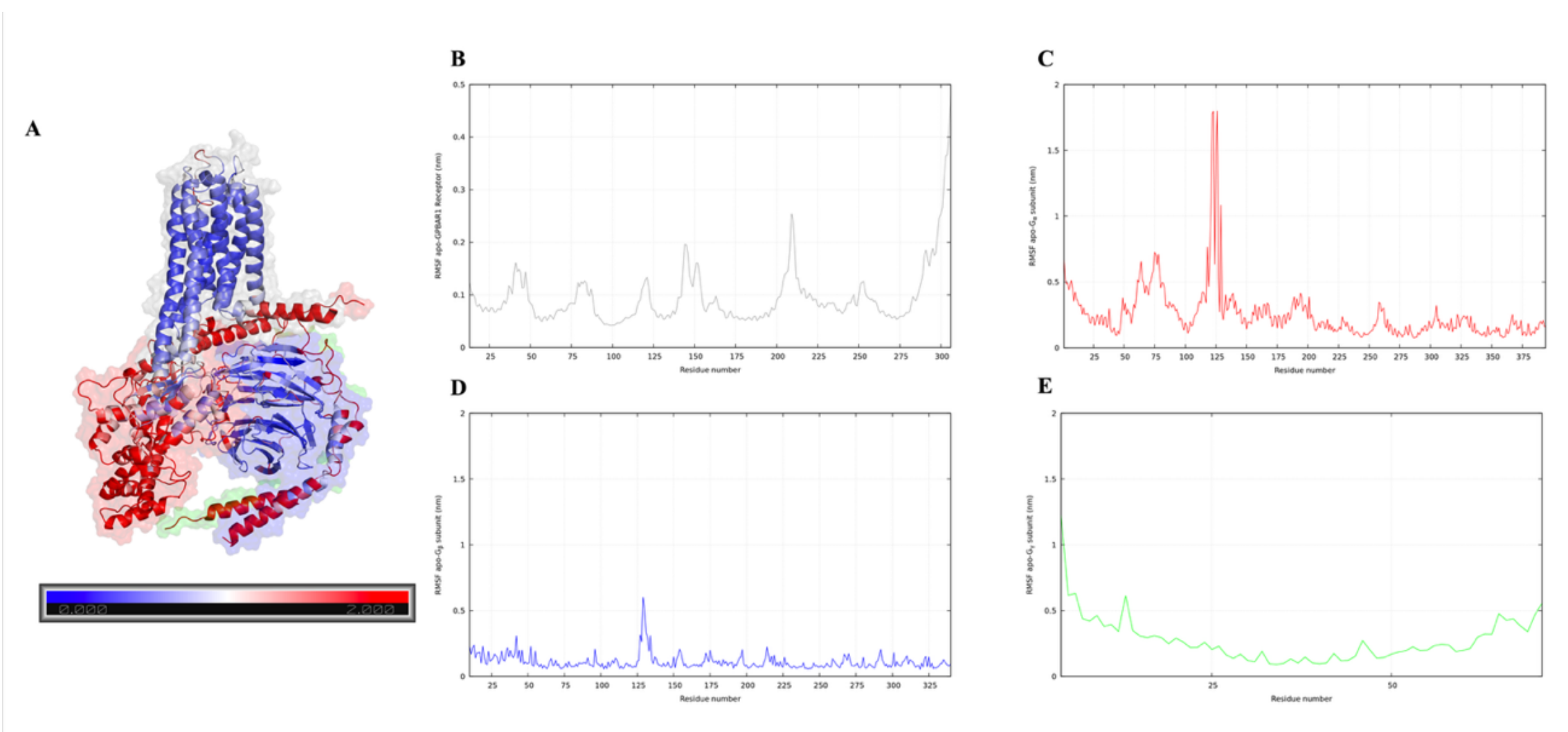

Figure S4. Root Mean Square Fluctuation (RMSF) analysis of the GPBAR1:G_s system computed over three independent 500 ns MD replicas. A) Structural representation of the GPBAR1:G_s complex colored according to the averaged RMSF-derived B-factors (scale 0-2). The intensity of the colors reflects the local structural flexibility: blue tones indicate low mobility, white intermediate mobility, and red tones high mobility of the backbone atoms. Panels B-E show the average RMSF profiles for: B) the GPBAR1 backbone; C) the Gα subunit backbone; D) the Gβ subunit backbone; and E) the Gγ subunit backbone. Higher RMSF values denote regions of increased structural mobility across the complex.

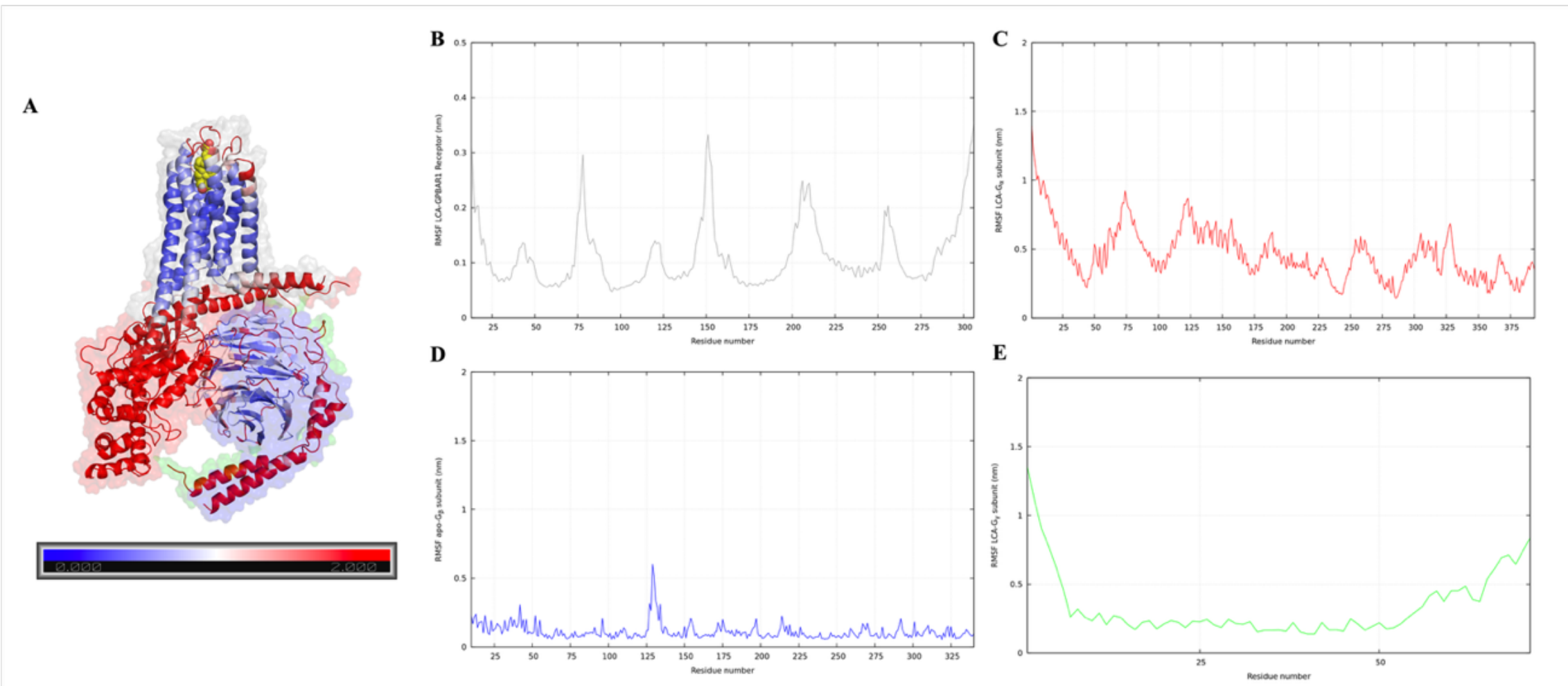

Figure S5. Root Mean Square Fluctuation (RMSF) analysis of the LCA:GPBAR1:G_s system computed over three independent 500 ns MD replicas. A) Structural representation of the LCA:GPBAR1:G_s complex colored according to the averaged RMSF-derived B-factors (scale 0-2). The intensity of the colors reflects the local structural flexibility: blue tones indicate low mobility, white intermediate mobility, and red tones high mobility of the backbone atoms; LCA is depicted as yellow spheres, with oxygen atoms in red. Panels B-E show the average RMSF profiles for: B) the GPBAR1 backbone; C) the Gα subunit backbone; D) the Gβ subunit backbone; and E) the Gγ subunit backbone. Higher RMSF values denote regions of increased structural mobility across the complex.

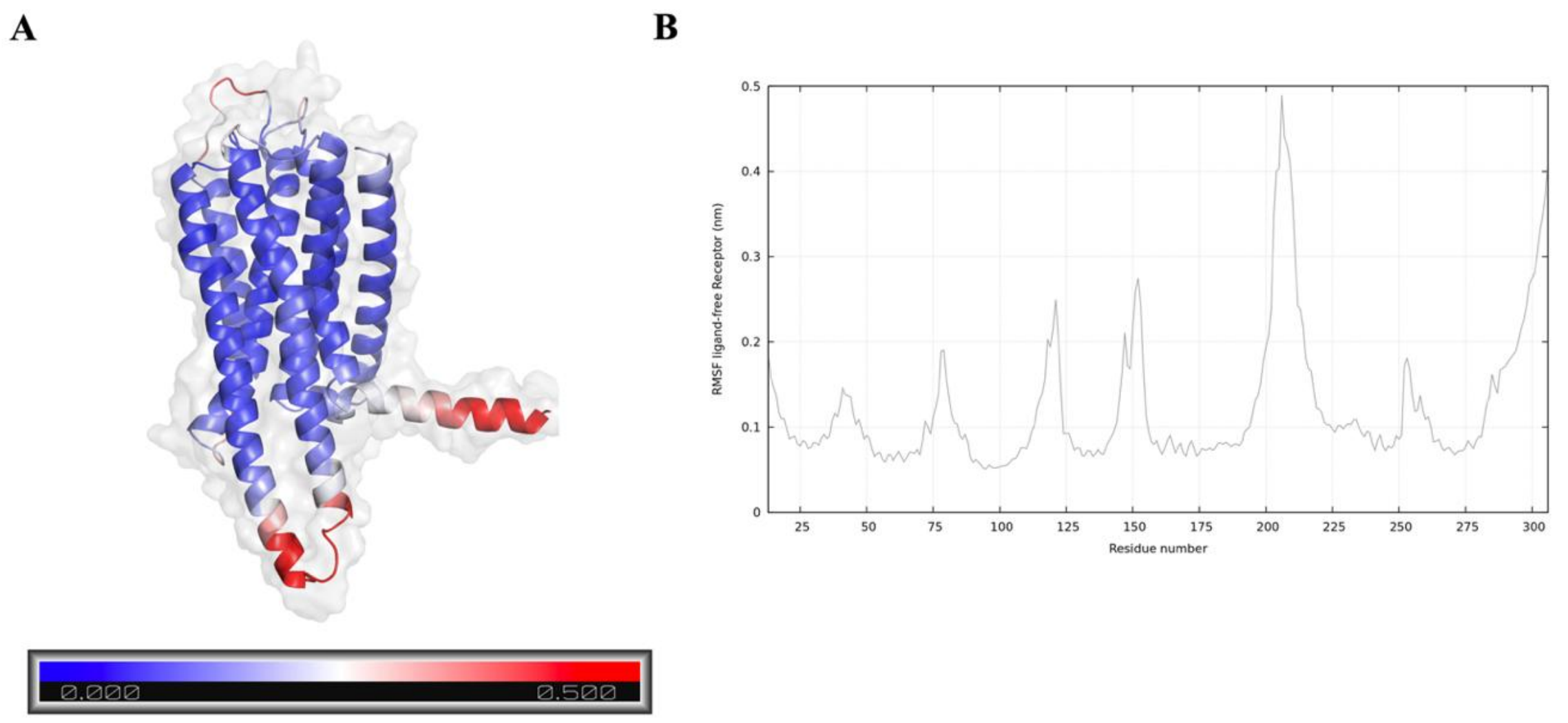

Figure S6. Root Mean Square Fluctuation (RMSF) analysis of the roGPBAR1 system computed over three independent 500 ns MD replicas. A) Structural representation of the roGPBAR1 receptor colored according to the averaged RMSF-derived B-factors (scale 0-0.5). The intensity of the colors reflects the local structural flexibility of the receptor backbone atoms: blue tones indicate low mobility, white intermediate mobility, and red tones high mobility. The reduced visualization scale compared to Figures S4 and S5 (scale 0–2) was adopted to better resolve local flexibility patterns within the G protein-free systems. B) Average RMSF profile calculated for the GPBAR1 receptor backbone. Higher RMSF values denote regions of increased structural flexibility across the receptor.

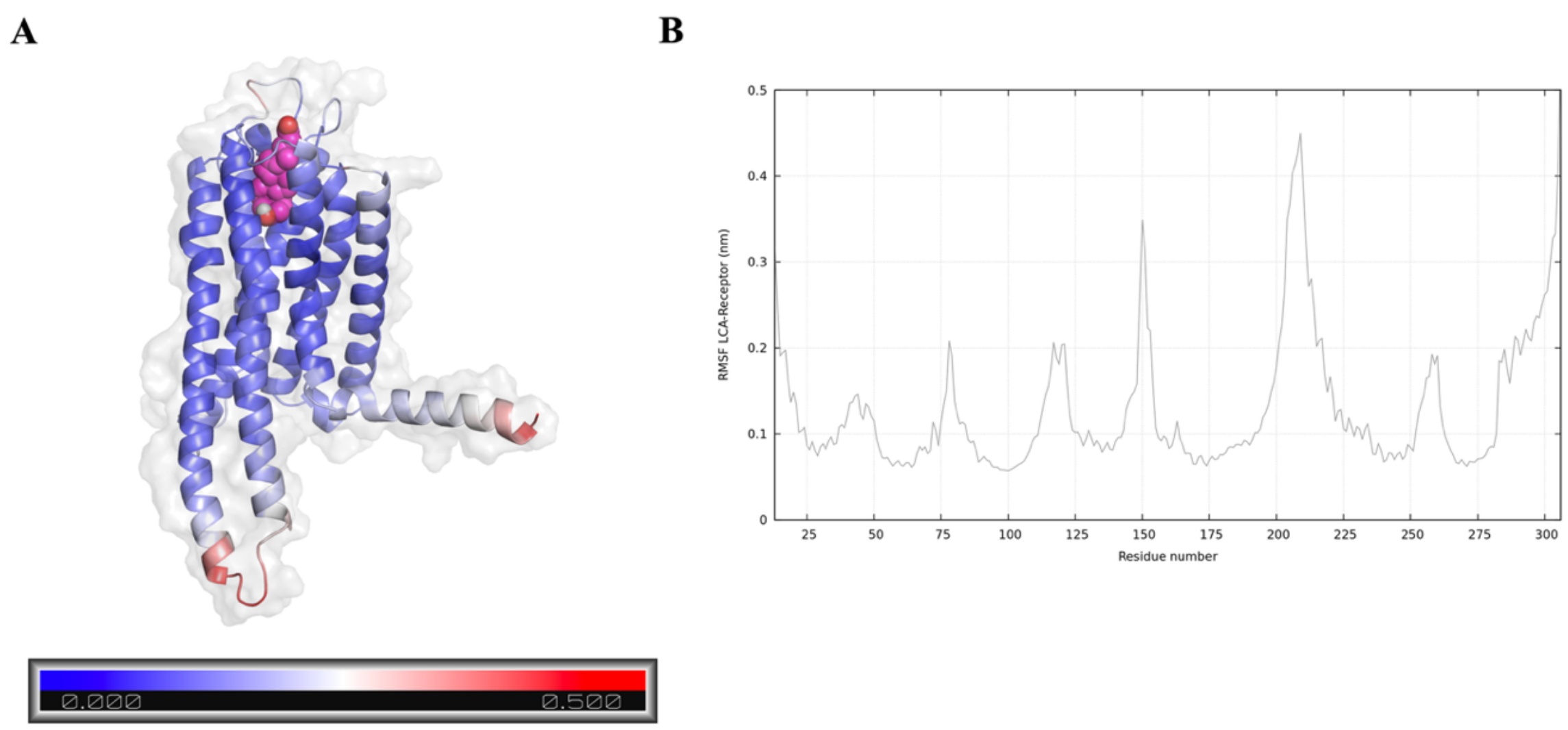

Figure S7. Root Mean Square Fluctuation (RMSF) analysis of the LCA:GPBAR1 system computed over three independent 500 ns MD replicas. A) Structural representation of

the LCA:GPBAR1 complex colored according to the averaged RMSF-derived B-factors (scale 0-0.5). The intensity of the colors reflects the local structural flexibility of the receptor backbone atoms: blue tones indicate low mobility, white intermediate mobility, and red tones high mobility; LCA is depicted as magenta spheres, with oxygen atoms in red. The reduced visualization scale compared to Figures S4 and S5 (scale 0–2) was adopted to better resolve local flexibility patterns within the G protein-free systems. B) Average RMSF profile calculated for the GPBAR1 receptor backbone. Higher RMSF values denote regions of increased structural flexibility across the receptor.

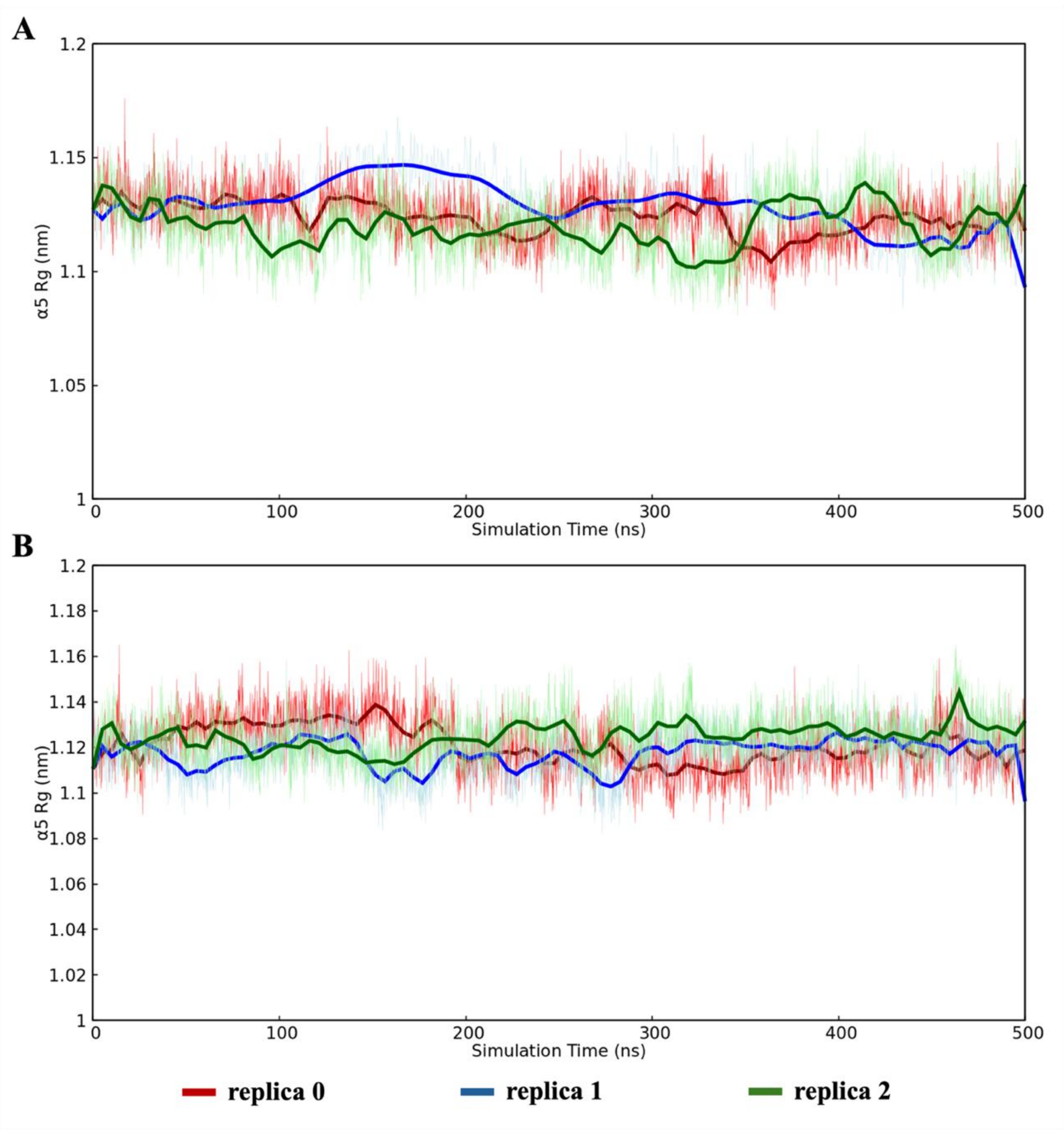

Figure S8. Radius of gyration (Rg) computed on the Gα_s α5-helix backbone over three independent replicas (500 ns each) in A) LCA:GPBAR1:G_s and B) GPBAR1:G_s systems. The red, blue and green traces correspond to replica 0, 1, and 2, respectively. Overall, both systems maintain a stable global compactness of the α5 helix throughout the simulations, with the LCA:GPBAR1:G_s complex displaying slightly enhanced fluctuations consistent with ligand-induced modulation of G-protein engagement and with transient partial unraveling and disengagement events of α5.

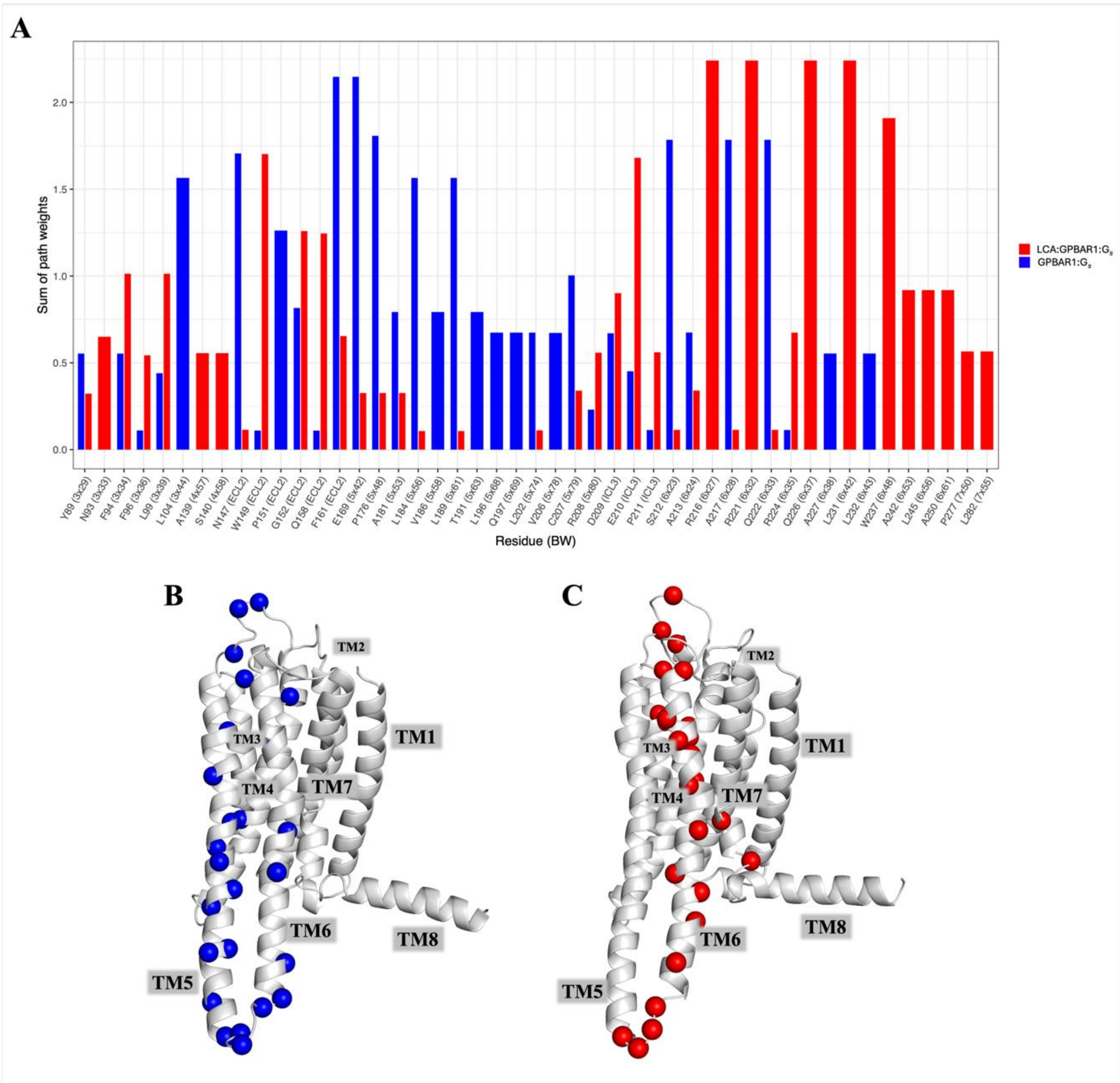

Figure S9. Allosteric communication pathways in GPBAR1 obtained from MDPath analysis. A) Plot of residues involved in allosteric communication; comparison of the

GPBAR1:G_s (panel B) and LCA:GPBAR1:G_s (panel C) states. Pathways are represented by Cα atoms shown as blue and red spheres for the GPBAR1:G_s and LCA:GPBAR1:G_s systems, respectively.

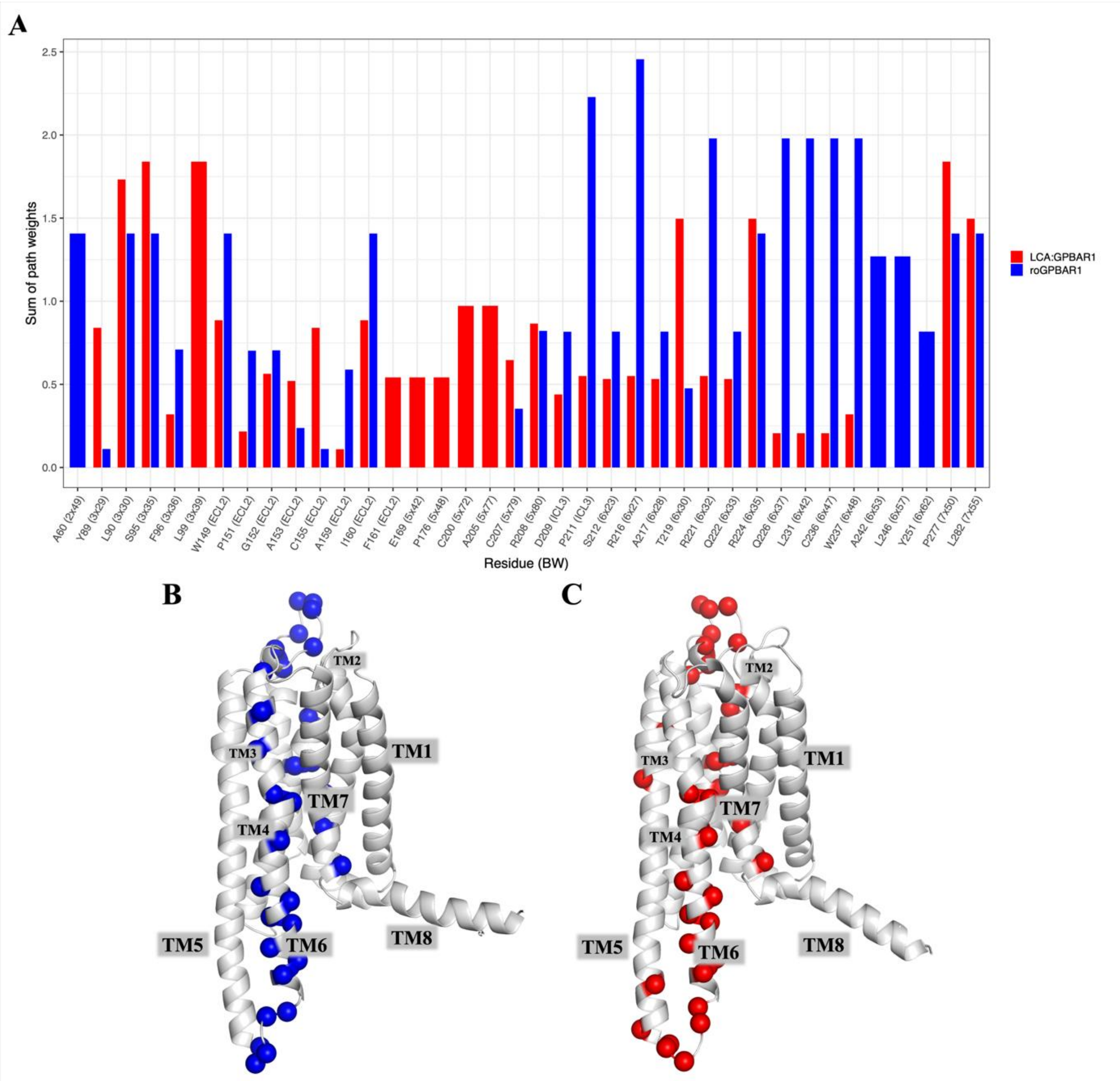

Figure S10. Allosteric communication pathways in GPBAR1 obtained from MDpath analysis. A) Plot of residues involved in allosteric communication; comparison of the roGPBAR1 (panel B) and LCA:GPBAR1 (panel C) states. Pathways are represented by Cα atoms shown as blue and red spheres for the roGPBAR1 and LCA:GPBAR1 systems, respectively.

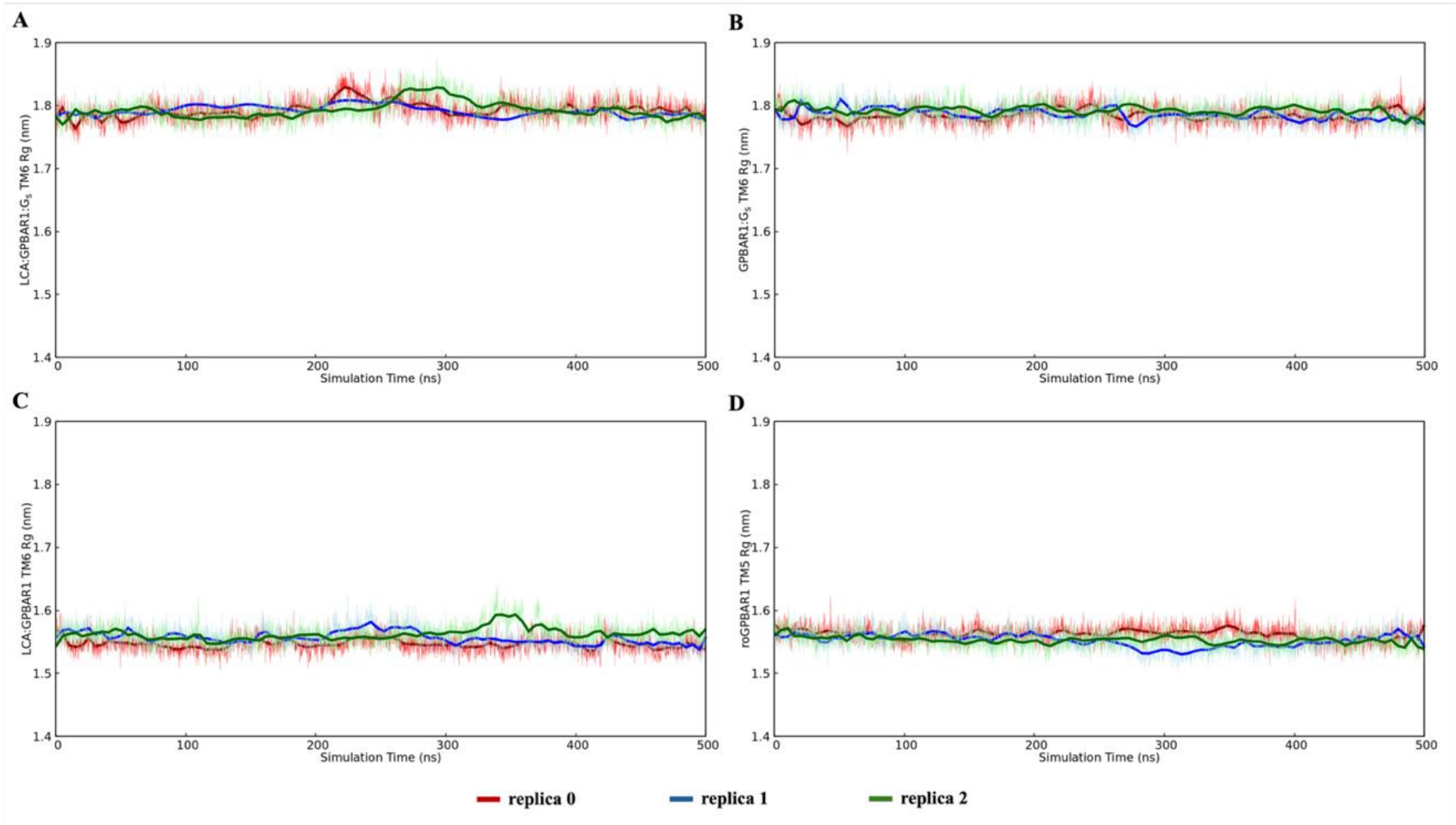

Figure S11. Radius of gyration (Rg) computed on the GPBAR1 receptor TM6 backbone over three independent replicas (500 ns each) in A) LCA:GPBAR1:G_s, B) GPBAR1:G_s, C) LCA:GPBAR1, and D) roGPBAR1. The red, blue and green traces correspond to replica 0, 1, and 2, respectively.

Table S1. Structural comparison of available GPBAR1 cryo-EM structures considered for template selection.

PDB ID	Release date	Resolution (Å)	Position	Ligand	RMSD compared to 7cfn
**7BW0**	12-04-2020	3.9	10-295	Ligand-free	1.032 Å (209 to 209 atoms)
**7CFM**	09-09-2020	3.00	20-292	Non steroidal	0.467 Å (232 to 232 atoms)
**7CFN**	09-09-2020	3.00	18-291	Steroidal	/
**7XTQ**	06-07-2022	3.20	20-292	Non-steroidal	0.435 Å (225 to 225 atoms)
**9GYO**	09-10-2024	2.50	20-292	Non-steroidal	0.356 Å (225 to 225 atoms)

Table S2. Cluster analysis of 1.5 μs MDs of GPBAR1:G_s system.

Cluster number	% of population	Average RMSD (nm)
1	33%	0.299
2	24%	0.287
3	13%	0.293
4	11%	0.304

Table S3. Cluster analysis of 1.5 μs MDs of LCA:GPBAR1:G_s system. Notably, the first two clusters calculated for the LCA:GPBAR1:G_s system were structurally similar, with a backbone RMSD of 0.16 nm.

Cluster number	% of population	Average RMSD (nm)
1	27%	0.300
2	26%	0.287
3	10%	0.314

Table S4. Cluster analysis of 1.5 μs MDs of roGPBAR1 system.

Cluster number	% of population	Average RMSD (nm)
1	12%	0.349

Table S5. Cluster analysis of 1.5 μs MDs of LCA:GPBAR1 system.

Cluster number	% of population	Average RMSD (nm)
1	25%	0.312
2	15%	0.301

Movie S1. Principal component analysis (PCA) of GPBAR1-$G\alpha_s\beta_1\gamma_2$ systems. The GPBAR1 receptor is shown as cyan cartoon, with TM5 and TM6 of the GPBAR1:G_s

system colored in blue, and the corresponding TM helices of the LCA:GPBAR1:G_s system in red. The movie illustrates the dominant conformational motions in the absence and presence of LCA.

Movie S2. Principal component analysis (PCA) of GPBAR1 systems. The GPBAR1 receptor is shown as cyan cartoon, with TM5 and TM6 of the roGPBAR1 system colored in blue, and the corresponding TM helices of the LCA:GPBAR1 system in red. The movie illustrates the dominant conformational motions in the absence and presence of LCA.
